# Effect of Acetic Acid and Lactic Acid at Low pH in Growth and Azole Resistance of *Candida albicans* and *Candida glabrata*

**DOI:** 10.3389/fmicb.2018.03265

**Published:** 2019-01-08

**Authors:** Andreia Lourenço, Nuno Alexandre Pedro, Sara Barbosa Salazar, Nuno Pereira Mira

**Affiliations:** Department of Bioengineering, Institute for Bioengineering and Biosciences, Instituto Superior Técnico, University of Lisbon, Lisbon, Portugal

**Keywords:** tolerance to acetic and lactic acids, vaginal candidiasis, Candida-bacteria interaction, *C. glabrata* and *C. albicans*, acetic and lactic acids

## Abstract

Successful colonization of the acidic vaginal niche by *C. glabrata* and *C. albicans* depends on their ability to cope with the presence of lactic and acetic acids produced by commensal microbiota. As such, the inhibitory effect of these acids at a low pH in growth of *C. glabrata* and *C. albicans* was investigated. The effect of the presence of these organic acids in tolerance of the two *Candida* species to azoles used in treatment of vaginal infections was also investigated including eventual synergistic effects. Under the different experimental conditions tested lactic acid exerted no significant inhibitory effect against *C. albicans* or *C. glabrata*, contrasting with the generalized impression that the production of this acid is on the basis of the protective effect exerted by vaginal lactobacilii. Differently, *C. glabrata* and *C. albicans* exhibited susceptibility to acetic acid, more prominent at lower pHs and stronger for the latter species. Synergism between acetic acid and azoles was observed both for *C. albicans* and *C. glabrata*, while lactic acid-azole synergism was only efficient against *C. albicans*. Altogether our *in vitro* results indicate that tolerance to acetic acid at a low pH may play a more relevant role than tolerance to lactic acid in determining competitiveness in the vaginal tract of *C. albicans* and *C. glabrata* including under azole stress. Treatment of vaginal candidiasis with azoles may depend on the level of acetic and lactic acids present and improvements could be achieved synergizing the azole with these acids.

## Introduction

*Candida* species are known commensals of the human genito-urinary and gastro-intestinal tracts however, under certain conditions, the colonizing *Candida* population can overgrow resulting in superficial infections or, in more serious cases, in life threatening disseminated mycoses. Vaginal infections are the leading superficial infections caused by *Candida* spp. being *C. albicans* and *C. glabrata* the top ethiological causative species(e.g., Sobel et al., 1998; Goncalves et al., 2016; Sobel, 2016). Vulvovaginal candidiasis (VVC) is estimated to affect 70–75% of all women, half of them experiencing at least one episode of re-incidence (Sobel, 2016). In more severe cases, the infection becomes recurrent giving origin to recurrent vulvovaginal candidiasis (RVVC), a condition that is estimated to affect around 138 million of women worldwide (Sobel, 2016). The high incidence and recurrence of vaginal candidiasis caused by *C. albicans* and *C. glabrata* results from these species being well-adapted to the vaginal environment rendering their eradication difficult (Sobel and Chaim, 1997; Sobel, 2007). A high adherence to vaginal epithelial cells mediated by specific adhesins, the ability to form biofilms on vaginal epithelium or in intra-uterine devices or the secretion of hydrolytic enzymes are among the factors attributed to vaginal pathogenicity of *Candida* spp. (Goncalves et al., 2016). High resilience to environmental stress and the natural increased resilience to azoles, the top therapeutic agents used in the treatment of superficial candidiasis, are distinguishable aspects of *C. glabrata* vaginal pathogenicity (Goncalves et al., 2016). The acidic environment of the vaginal tract (pH in the range of ~4–4.5 (Aldunate et al., 2015) was suggested to contribute for the increased resilience to azoles of *C. glabrata* and *C. albicans* based on the demonstrated decreased efficacy of these drugs *in vitro* at acidic pHs (Danby et al., 2012; Kasper et al., 2015; Boikov et al., 2017; Spitzer and Wiederhold, 2018). In these studies the acidic pH of the vaginal tract was mimicked adjusting the pH of the medium with a strong acid, however, vaginal acidity results from the presence of lactic and acetic acid that are produced by epithelial cells and/or by the co-colonizing microbiota (Boskey et al., 1999, 2001; Aldunate et al., 2015). While organic acids are able to dissociate directly in the near neutral microbial cytosol due to the lipophilic properties of the undissociated acid form, chemical dissociation of strong acids results in the accumulation of protons in the environment which do not cross the cell envelope at a significantly extent due to their charge (Mira et al., 2010). This ability to dissociate directly inside microbial cells turns weak organic acids much more efficient as antimicrobials, comparing with strong acids (Mira et al., 2010). Besides inducing intracellular acidification, exposure to weak organic acids also results in internal accumulation of the negatively charged counter-ion leading to multiple deleterious effects for yeast cells that include, an increased turgor pressure, oxidative stress, depletion of ribosomal RNA, or of relevant cofactors, among others (Mira et al., 2010). Although much of these effects have been studied in the experimental model yeast *Saccharomyces cerevisiae*, more recent studies undertaken in *C. albicans* and in *C. glabrata* confirm similar toxicity mechanisms (Cottier et al., 2015, 2017; Bernardo et al., 2017; Cunha et al., 2017).

Although the presence of organic acids is largely recognized as an hallmark of vaginal health and a key factor in preventing overgrowth of pathogens, including of *Candida* spp. (Yan et al., 2009; Hickey et al., 2012), the eventual inhibitory effect of these molecules in inhibiting growth of *Candida* species has not been examined in a systematic and comparative manner including the study of eventual synergistic effects between the two acids. The effect exerted by these molecules in modulating tolerance of *Candida* spp. to azoles has also only been poorly studied. A study undertaken by Moosa et al. (2004) has shown that acetic acid (but not lactic acid) is able to potentiate the activity of fluconazole against *C. albicans*, however, in this work only one concentration of acetic or lactic acids has been used reflecting the concentration expected in vaginal secretions in healthy nonpregnant premenopausal women (Moosa et al., 2004). Differently, in our study we have used a range of concentrations of the two organic acids observed to occur in the vaginal tract under different conditions thus better reflecting the changing environment that *Candida* species are challenged in this infection site. Another distinctive aspect of our study is the use of other topical azoles besides fluconazole and also the focus on *C. glabrata* species and on the use of azole-resistant strains of this species which are herein shown to be sensitized in the presence of acetic acid.

## Materials and Methods

### Strains and Growth Media

The strains used in this study are described in Table 1. The *Candida* strains used were batch-cultured at 30°C, with orbital agitation (250 rpm) in minimal medium (MM), in rich yeast peptone dextrose (YPD) or in RPMI. MM contains, per liter, 1.70 g yeast nitrogen base (YNB) without amino acids and NH4^+^ (Difco Laboratories, Detroit, Mich.), 2.65 g (NH_4_)_2_SO4 (Merck Millipore) and glucose (10 or 2 g/L) (Merck Millipore, Darmstadt, Germany). YPD contains, per liter, 20 g glucose (Merck Millip3ore), 10 g yeast extract (HiMedia Laboratories, Mumbai, India) and 20 g peptone (HiMedia Laboratories). RPMI (Roswell Park Memorial Institute Medium) contains, per liter, 10.8 g RPMI-1640 synthetic medium (Sigma), 18 g glucose (Merck Millipore), and 34.5 g of MOPS (3-(N-morpholino) propanesulfonic acid, Sigma). When required the pH of the different growth media was adjusted using HCl or NaOH. All media were prepared in deionized water and sterilized by autoclaving for 15 min at 121°C and 1 atm, except RPMI that was sterilized by filtration. Solid media were obtained by supplementing the corresponding liquid growth medium with 20 g (per liter) of agar (Iberagar). The pH of the acetic and lactic acid stock solutions used were adjusted to pH 4.0 using NaOH 10 M and/or HCl. The stock solutions of the antifungals, clotrimazole, miconazole, fluconazole and tioconazole were prepared from the powder and using DMSO (Dimethyl sulfoxide, Sigma) as the solvent. All antifungals were purchased from Sigma.

**Table 1:** Strains of *Candida* species used in this study.

**Strain**	**Description**	**References**
*C. albicans* SC5314	Reference strain	-
*C. albicans* VG216	Vaginal clinical isolate	This study
*C. albicans* VG485	Vaginal clinical isolate	This study
*C. glabrata* CBS138	Reference strain	Dujon et al., 2004
*C. glabrata* BG2	Vaginal clinical isolate	Fidel et al., 1996
*C. glabrata* VG99	Vaginal clinical isolate	Cunha et al., 2017
*C. glabrata* VG281	Vaginal clinical isolate	Cunha et al., 2017
*C. glabrata* VG216	Vaginal clinical isolate	Cunha et al., 2017
*C. glabrata* FFUL887	Azole-resistant clinical isolate harboring a gain-of-function CgPdr1 allele (K274Q substitution)	Salazar et al., 2018
*C. glabrata* FFUL667	Azole-resistant clinical isolate	Salazar et al., 2018
*C. glabrata* FFUL674	Azole-resistant clinical isolate harboring a gain-of-function CgPdr1 allele (I803T substitution)	Salazar et al., 2018
*C. glabrata* F15	Azole-resistant clinical isolate harboring a gain-of-function CgPdr1 allele (P927L substitution)	Vermitsky et al., 2006

### Assessment of Susceptibility to Acetic and/or Lactic Acids

The susceptibility of *C. albicans* SC5314, *C. glabrata* CBS138 and *C. glabrata* BG2 to acetic and lactic acids was tested in 96-well microplates containing MM medium (having 1% or 0.2% glucose) either or not supplemented with acetic or lactic acids. Five concentrations of each acid were tested: 0.4, 4, 30, 45, and 75 mM for acetic acid and 80, 100, 120, 140, 160 mM for lactic acid. The media and the organic acids concentrations were adjusted to pH 4.5, 4, 3.5, and 3, always using HCl as the acidulant. Mid-exponential phase cells (OD_600nm_ of ~0.8) cultivated in MM medium were used to inoculate the 96-microwell plates at an initial OD_600nm_ of 0.05. The inoculated plates were incubated at 30°C (using an agitation of 20 rpm or 200 rpm) for 24 h and growth was accompanied based on the increase in OD_600nm_. The exact same experimental setting was used to assess susceptibility of the different *Candida* strains to acetic or lactic acids in RPMI medium (having 2 or 0.2% glucose). For the synergistic assays the same experimental setting was used with the difference that the MM medium (at pH 4) was supplemented with both acetic and lactic acids (the same concentration range was used). All assays were performed in triplicates.

### Assessment of Susceptibility to Azoles

Susceptibility of *C. glabrata* CBS138*, C. glabrata* BG2 and *C. albicans* SC5314 to clotrimazole, fluconazole, miconazole, and tioconazole in the presence of lactic or acetic acids was tested using a similar setup to the one described above. The concentrations of antifungals used were: 1, 5, 7.5, and 10 mg/L for clotrimazole; 30, 60, 64, and 128 mg/L for fluconazole; 0.05, 0.2, and 0.4 mg/L for miconazole and 0.1, 0.3, 0.45, and 0.6 mg/L for tioconazole. In all cases the stock solution of the antifungal was adjusted at pH 4. The concentrations of acetic acid tested were 4, 40, and 75 mM for acetic acid, while for lactic acid it were tested 80, 120, and 160 mM. Synergism between the two acids was only considered when the reduction in growth obtained in the medium supplemented with the organic acid and the azole drug was 50% higher than the inhibition registered when the cells were incubated with the compounds individually. In the case of fluconazole and clotrimazole it was also determined the MIC50 for the above referred *Candida* strains as well as for the clinical isolates *C. albicans* VG216, *C. albicans* VG485, *C. glabrata* FFUL887, *C. glabrata* VG99, *C. glabrata* VG216, *C. glabrata* VG281, *C. glabrata* 674, and *C. glabrata* 677. For this the microdilution method was used as recommended by EUCAST (Subcommittee on Antifungal Susceptibility Testing (AFST) of the ESCMID European Committee for Antimicrobial Susceptibility Testing (EUCAST) et al., 2003). All assays were performed in quadruplicates.

## Results

### *At a Low pH, C. glabrata* and *C. albicans* Are Susceptible to Acetic Acid but Not to Lactic Acid

**Figure 1:**
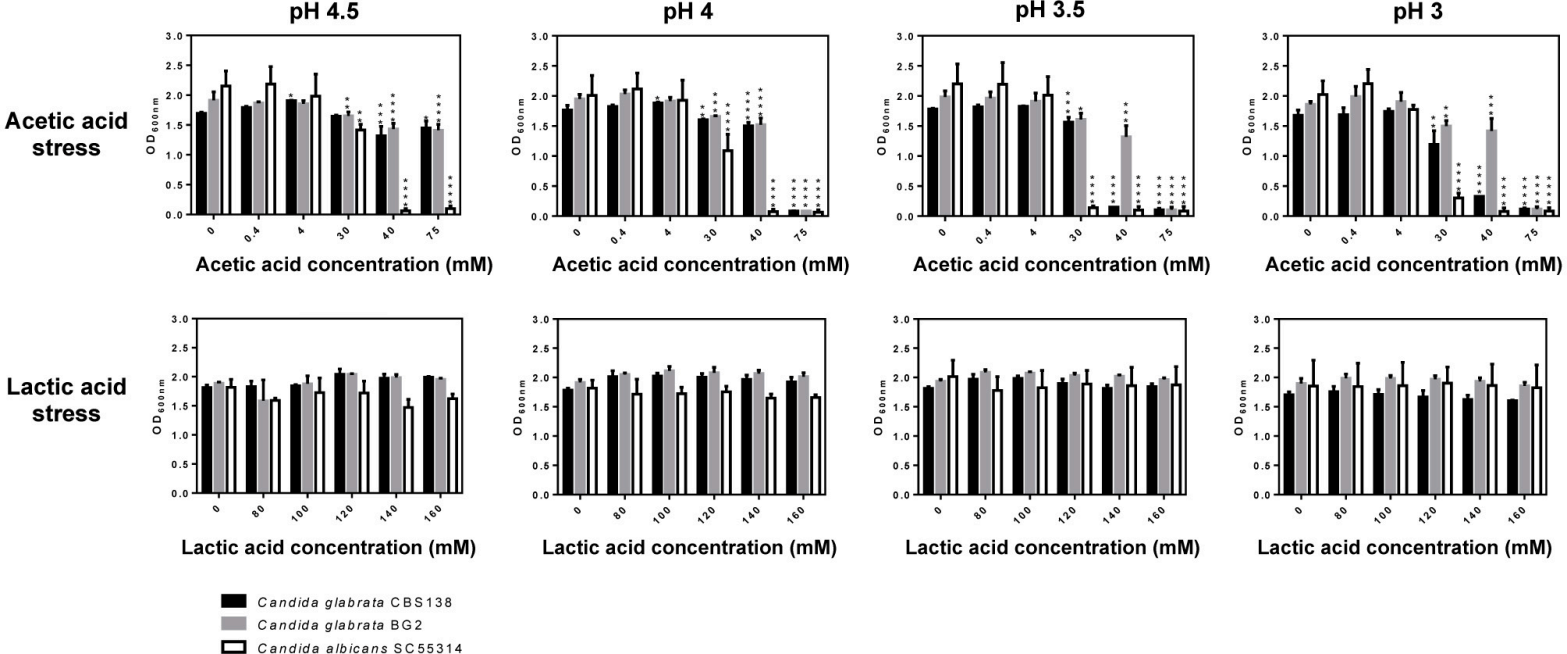
Growth under aerophilic conditions of *C. albicans* (white bars), *C. glabrata* CBS138 (black bars) and *C. glabrata* BG2 (gray bars) in MM medium supplemented with the indicated concentrations of lactic or acetic acid at the pHs depicted in the figure. Statistical significance was calculated comparing growth of each strain in the presence and absence of acetic or lactic acids (^*^p-value below 0.05; ^**^p-value below 0.01; ^***^p-value below 0.001; ^****^p-value below 0.0001).

**Figure 2:**
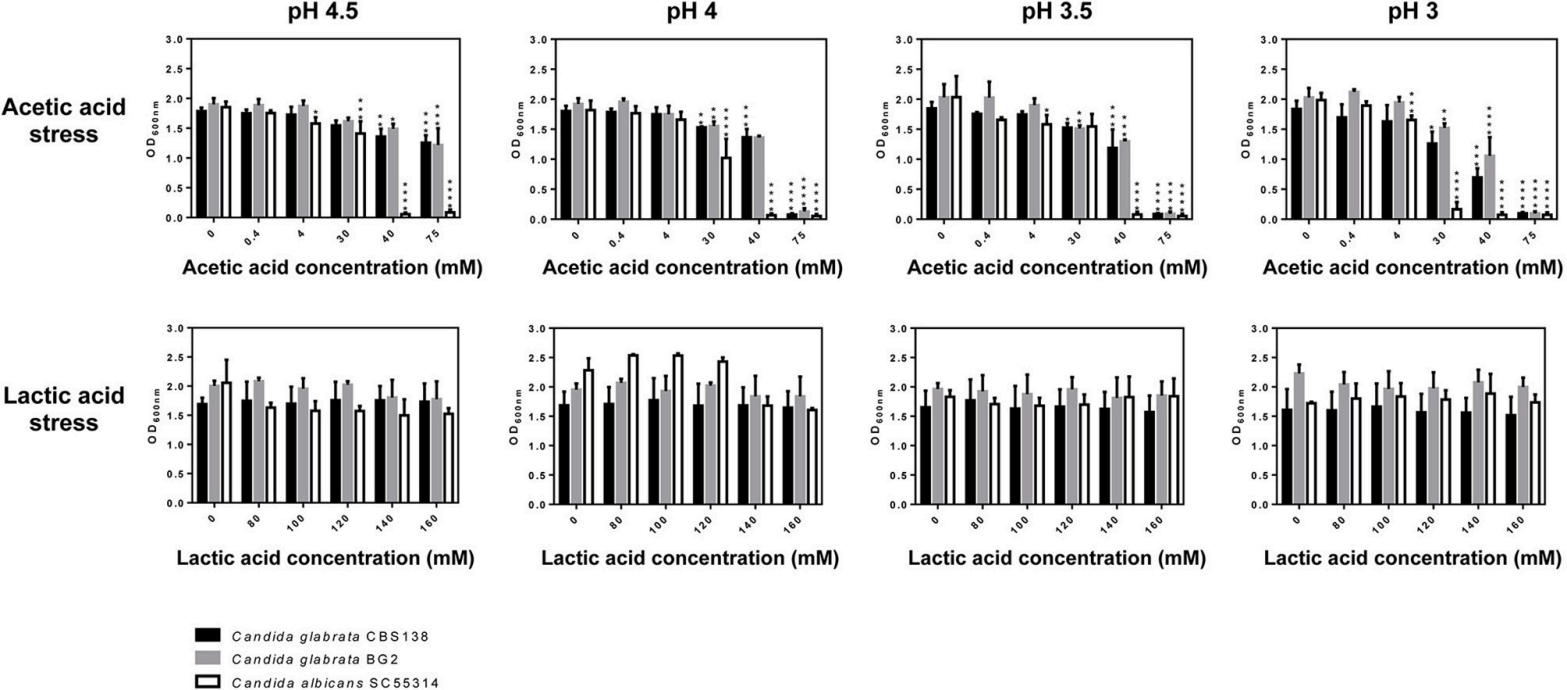
Growth under microaerophilic conditions of *C. albicans* (white bars), *C. glabrata* CBS138 (black bars) and *C. glabrata* BG2 (gray bars) in MM medium supplemented with 0.2% glucose and with the indicated concentrations of lactic or acetic acid at the pHs depicted in the figure. Statistical significance was calculated comparing growth of each strain in the presence and absence of acetic or lactic acids (^*^p-value below 0.05; ^**^p-value below 0.01; ^***^p-value below 0.001; ^****^p-value below 0.0001).

*C. glabrata* and *C. albicans* cells are challenged with acetic (1–120 mM) and lactic acids (~120 mM) (Boskey et al., 1999, 2001; Aldunate et al., 2015) at a low pH in the acidic vaginal tract. Thus, we have monitored growth of these two pathogenic species in MM medium supplemented with increasing concentrations of the two organic acids (in the range of 4–75 mM for acetic acid and 80–160 mM for lactic acid) at pHs ranging from 3 to 4.5 (Figure 1). The reference strain *C. albicans* SC5314 and two *C. glabrata* laboratory strains: the reference strain CBS138 and BG2, used to study vaginal pathophysiology of *C. glabrata* (Fidel et al., 1996), were used. The results obtained clearly demonstrate that lactic acid exerts very little effect against the three strains tested, not even at the lower pH tested of 3 (Figure 1). Consistently, the number of viable *C. albicans* SC5314 and *C. glabrata* CBS138 recovered from cells cultivated for 24h in presence of 160 mM lactic acid at pH 4 was only slightly below the levels attained in control cells (Figure S1). Differently, in the presence of the higher concentrations of acetic acid tested (40 and 75 mM) a prominent reduction in growth of the two *Candida* species tested was observed, this being significantly more marked for *C. albicans* (Figure 1). As expected, the toxic effect of acetic acid was augmented with the reduction in pH (Figure 1). Consistent with the growth inhibition observed, a significant loss of cellular viability (of around 100-fold) was observed in the acetic acid stressed (75 mM at pH 4) *C. albicans* and *C. glabrata* populations (Figure S1). The inhibition in growth caused by acetic acid cannot be attributable to the acidification of the medium itself since acidification using the strong acid HCl as the acidulant agent did not led to growth inhibition for any of the strains tested (results not shown). Under the conditions that we have used no significant filamentation of *C. albicans* cells was observed (as assessed by microscopic observation of culture samples) (results not shown), in line with the previous demonstration that morphological yeast-hyphae transition does not occur at a significant extent in acidic pHs (Davis et al., 2000). The high tolerance of the two *C. glabrata* strains tested to acetic and lactic acids is in line with the described high resilience of this species to environmental stress (Jandric and Schuller, 2011). The vaginal strain *C. glabrata* BG2 showed higher tolerance against acetic acid than the reference strain CBS138, a trait that could be attributable to the development of efficient adaptive responses to cope with acetic acid stress at a low pH by vaginal *C. glabrata* strains (Cunha et al., 2017). The amount of glucose in the vaginal environment is low (around 0.5%) (Owen and Katz, 1999; Childers et al., 2016) and thus the phenotypic screening was repeated using the same mineral medium but having only 0.2% glucose (Figure S2). The results obtained were essentially the same as those shown in Figure 1, only being detectable a toxic effect against *C. albicans* and *C. glabrata* exerted by acetic acid (Figure S2). The same results were also obtained when RPMI was used (Figure S3). Since the vaginal environment is microaerophilic (Sosinska et al., 2008) we have also examined the toxic effect imposed by acetic or lactic acids at a low pH in growth of *C. albicans* and *C. glabrata* under those conditions (Figure 2 and Figures S4, S5). Although a generalized reduced growth in the O_2_-limited setting was observed, the results obtained concerning the effect of acetic acid and the non-inhibition caused by lactic acid were similar (Figure 2 and Figures S4, S5). We have also tested whether the increase in temperature from 30° to 37°C would change the pattern of acetic and lactic-susceptibilities observed, however, the results obtained at the two temperatures were identical (Figure S6).

### At Low pH Lactic and Acetic Acids Do Not Synergistically Inhibit Growth of *Candida* spp.

**Figure 3:**
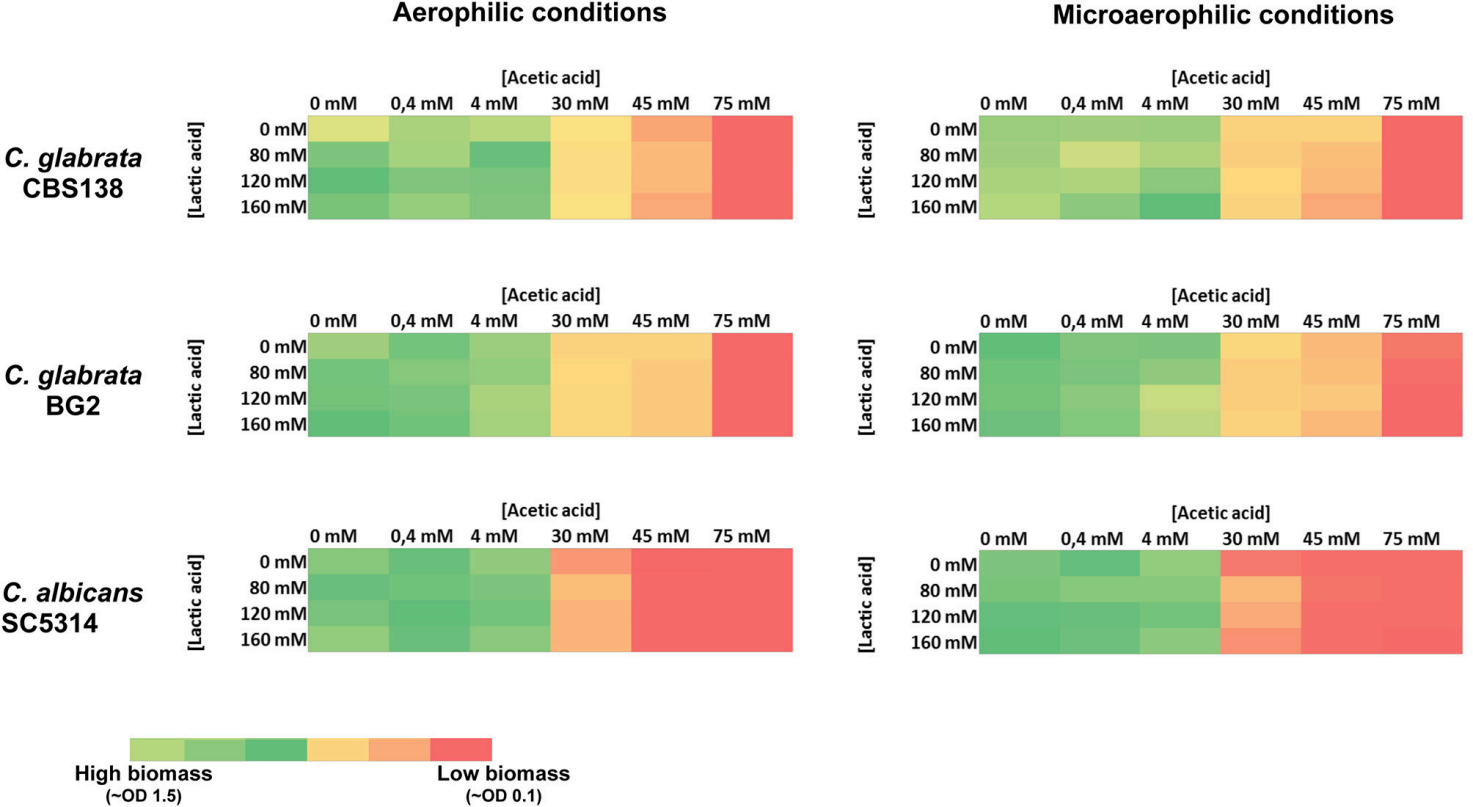
Heat map representing the inhibitory effect of lactic and/or acetic acids against *C. albicans, C. glabrata* BG2 and *C. glabrata* CBS138 in MM medium (at pH 4). To assess an eventual synergistic effect of lactic and acetic acids in determining growth inhibition of *Candida* spp. growth of the different strains in MM medium (at pH4) in the presence of different concentrations of these organic acids was assessed based on the OD600nm after 24 h of incubation in the presence of the acids, which was used to build the heat-map depicted.

Since acetic and lactic acids exist together in the vaginal tract, an eventual synergistic effect between these two acids in inhibiting growth of *C. albicans* and *C. glabrata* was hypothesized. To test this, the strains were cultivated under the same experimental conditions described above with the difference that this time both acetic and lactic acids were simultaneously added to the growth medium. The results obtained (shown in Figure 3) confirmed the lack of toxicity of lactic acid, while exposure to acetic acid resulted in growth inhibition, specially for concentrations above 40 mM (Figure 3). The hypothesized synergism between the two acids was not confirmed since the presence of lactic acid did not augmented the strong toxic effect exerted by acetic acid at pH 4. A similar result was also obtained in medium containing lower concentrations of glucose (0.2% instead of 1%) (results not shown) or under microaerophilic conditions (Figure 3).

### The Presence of Acetic and Lactic Acids at Low pH Modulates Tolerance to Azoles in *C. albicans and C. glabrata*

**Figure 4:**
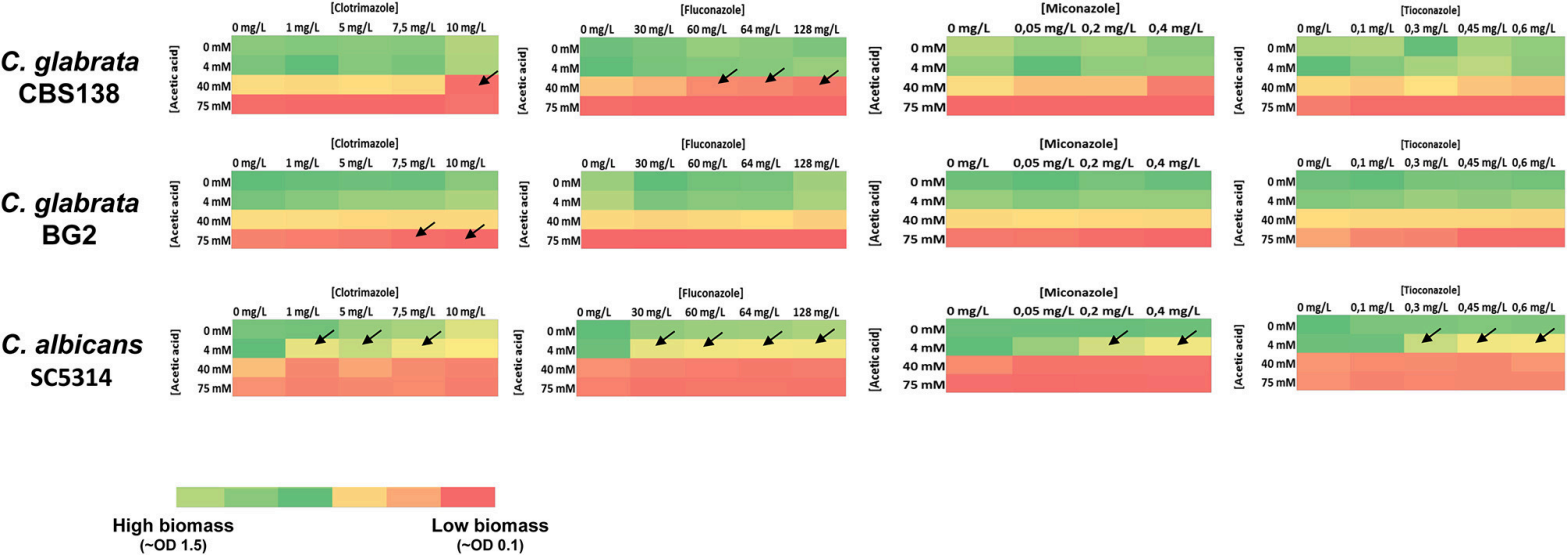
Heat map representing the inhibitory effect of acetic acid and clotrimazole, fluconazole, miconazole, and tioconazole against *C. albicans, C. glabrata* BG2 and *C. glabrata* CBS138 in MM medium (at pH 4). To assess an eventual synergistic effect of acetic acids with the different azoles in determining growth inhibition of *Candida* spp., growth of the different strains in MM medium (at pH4) in the presence of different concentrations of acetic acid and of the different azoles was assessed based on the OD600nm after 24 h of incubation in the presence of the acids, as detailed in Materials and Methods. Synergism between acetic acid and an azole was only considered when growth inhibition achieved in the presence of the acid and of the azole was, at least, 50% higher the inhibition observed in the presence of the azole alone or of the acid alone. Conditions where the acid and an azole were found to synergize are represented by an arrow. Details on the results obtained in those conditions where azoles and acetic acid were found to synergize are provided in Supplementary Figure S7 (^*^p-value below 0.05; ^**^p-value below 0.01; ^***^p-value below 0.001; ^****^p-value below 0.0001).

**Figure 5:**
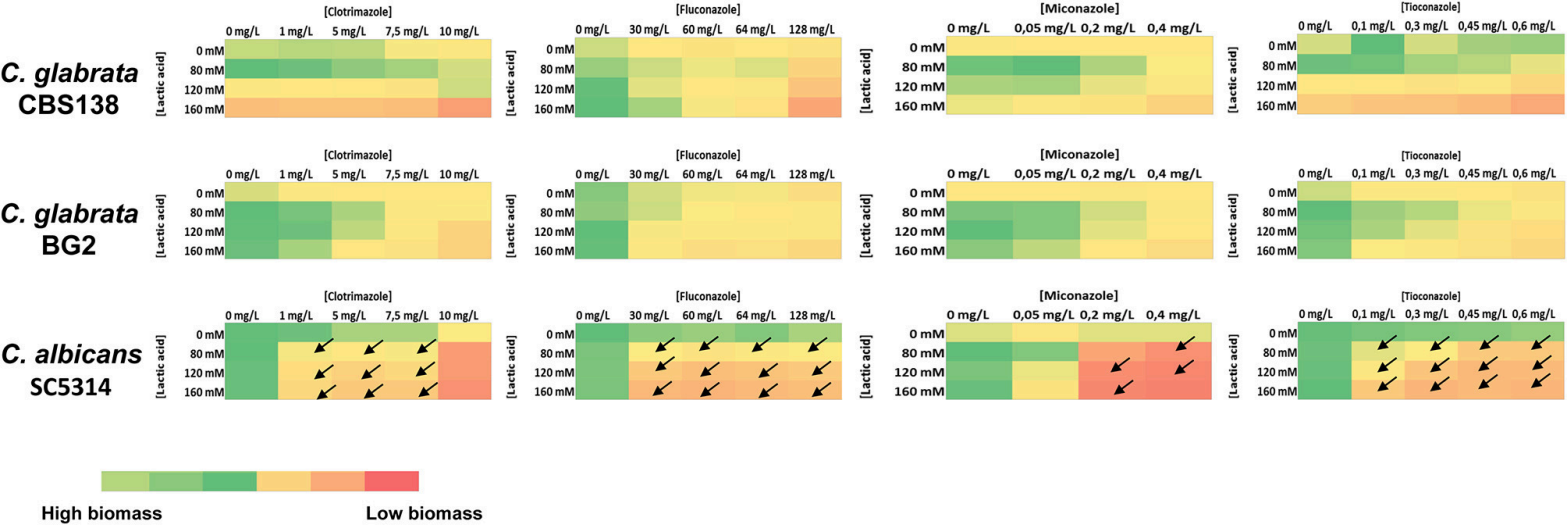
Heat map representing the inhibitory effect of lactic acid and clotrimazole, fluconazole, miconazole, and tioconazole against *C. albicans, C. glabrata* BG2 and *C. glabrata* CBS138 in MM medium (at pH 4). To assess an eventual synergistic effect of lactic acid with the different azoles in determining growth inhibition of *Candida* spp., growth of the different strains in MM medium (at pH4) in the presence of different concentrations of acetic acid and of the different azoles was assessed based on the OD600nm after 24 h of incubation in the presence of the acids, as detailed in Materials and Methods. Synergism between lactic acid and an azole was only considered when growth inhibition achieved in the presence of the acid and of the azole was, at least, 50% higher the inhibition observed in the presence of the azole alone or of the acid alone. Conditions where the acid and an azole were found to synergize are represented by an arrow. Details on the results obtained in those conditions where azoles and lactic acid were found to synergize are provided in Supplementary Figure S7.

Since vaginal candidiasis is typically treated using topical azoles we have tested whether the presence of acetic and lactic acids at a low pH could influence the activity of clotrimazole, miconazole, tioconazole, and fluconazole. For that, cells were cultivated in MM growth medium (having 1% glucose and adjusted at pH 4) supplemented with inhibitory concentrations of the different azoles and/or with acetic (Figure 4) or lactic (Figure 5) acids. Acetic acid synergized with clotrimazole and fluconazole in inhibiting growth of *C. glabrata* and *C. albicans* (Figure 4 and Figure S7). It has to be pointed out, however, that for the *C. glabrata* BG2 strain the synergistic effect was only observed for the higher concentration of acetic acid tested, 75 mM, most likely due to the higher resilience of this strain to acetic acid. A synergistic effect with miconoazole and thioconazole was also observed but only against *C. albicans* (Figure 4 and Figure S7). Lactic acid synergized with all the tested antifungals but only against *C. albicans* (Figure 5 and Figure S7).

**Figure 6:**
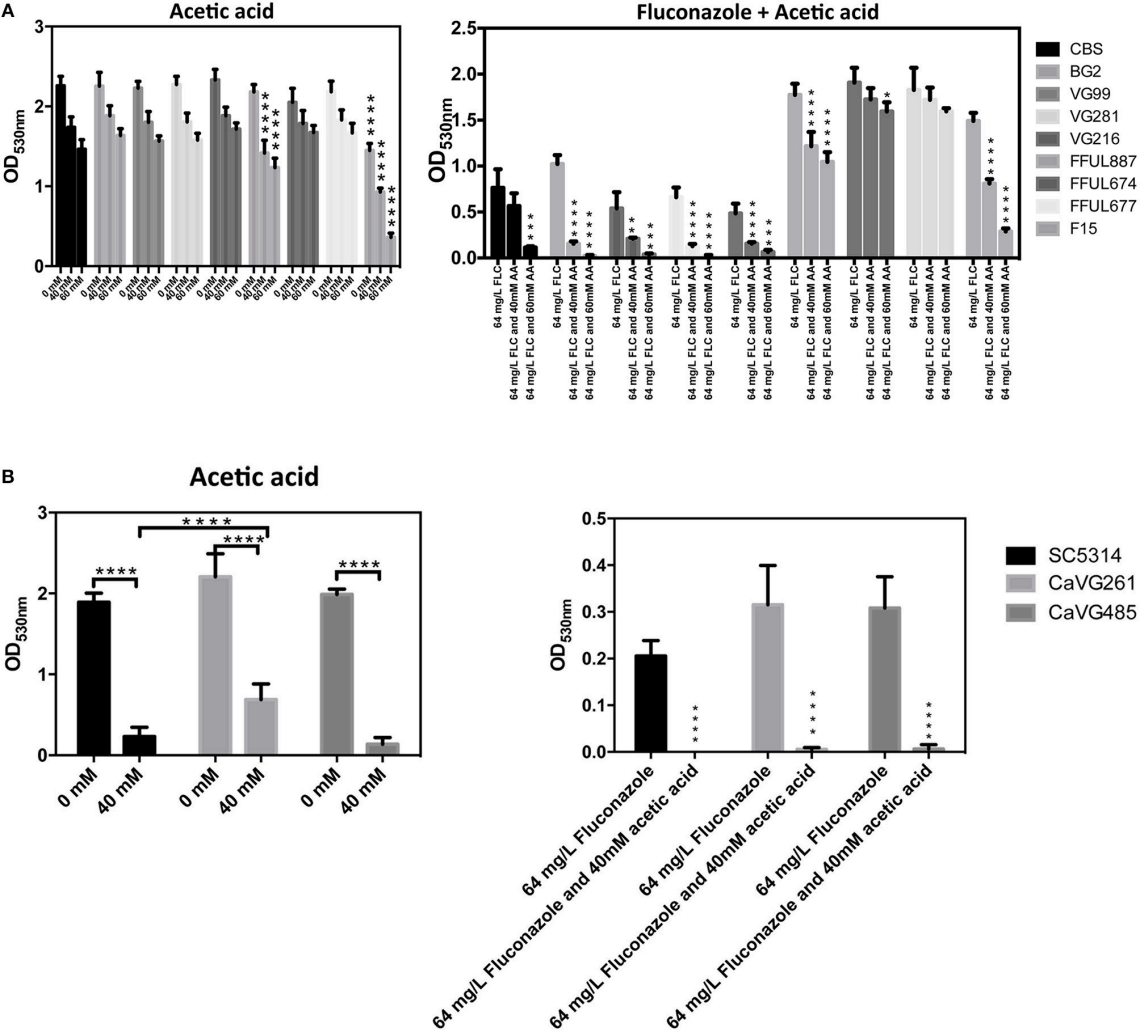
Susceptibility of *C. glabrata* **(A)** and *C. albicans* **(B)** strains to acetic acid, to fluconazole or to a combination of fluconazole and acetic acid. This picture depicts the final OD600nm of cultures of the different strains used in our study after 24 h of cultivation in RPMI medium supplemented with acetic acid (40 or 60 mM, at pH 4) or in this same medium supplemented with fluconazole (64 mg/L) or with fluconazole and acetic acid (^*^p-value below 0.05; ^**^p-value below 0.01; ^***^p-value below 0.001; ^****^p-value below 0.0001).

The observed synergistic effects between acetic and/or lactic acid with antifungals of clinical application prompted us to test whether the presence of the organic acids would alter the MIC level of the strains, as determined by the micro-dilution method recommended by EUCAST. Besides using reference, we have also used three *C. glabrata* vaginal strains (VG99, VG281, and VG216), two *C. albicans* vaginal strains (CaVG674 and CaVG677) and four previously documented *C. glabrata* azole-resistant strains (FFUL887, FFUL674, FFUL677, and F15) (Vermitsky et al., 2006; Salazar et al., 2018). The assays were conducted at two distinct pHs (4 and 7) to tackle the effect of the undissociated form of the acid in the eventual modulation of azole resistance of the strains. The results obtained confirm the previously reported reduction of fluconazole and clotrimazole efficacy in inhibiting growth of *C. albicans* and *C. glabrata* at acidic pHs when strong acids are used as acidulants (Danby et al., 2012), this being observed for all the strains tested (Table 2). In the presence of acetic acid at pH4 the susceptibility of the more azole-susceptible strains (*C. glabrata* CBS138, BG2, VG281, VG99, and VG16 and *C. albicans* SC5314, CaVG674, and CaVG677) to fluconazole and clotrimazole was significantly enhanced being observed a prominent reduction in the MIC level of the strains (Table 2). Within the cohort of four azole-resistant *C. glabrata* strains herein examined, the F15 strain exhibited a prominent increase in susceptibility to fluconazole and clotrimazole (F15) in the presence of acetic acid (at pH 4) while for strains FFUL887 and FFUL677 a reduction in growth was also observed (Figures 6A,B), although this was not enough to cause a reduction in the MIC (Table 2). The observed increase in susceptibility of the strains to azoles in the presence of acetic acid correlated with their increased susceptibility to acetic acid alone (Figures 6A,B).

**Table 2:** MIC for fluconazole (FLC) and clotrimazole (CLTRI) in the presence or absence of acetic acid (AcA, 40, and 60 mM) for several *C. glabrata* and *C. albicans* strains.

	**pH 4**						**pH 7**					
**Strain**	**MIC FLC (mg/mL)**	**MIC FLC (in the presence of 40 mM AcA) (mg/mL)**	**MIC FLC (in the presence of 60 mM AcA) (mg/mL)**	**MIC CLTRI (mg/mL)**	**MIC CLTRI (in the presence of 40 mM AcA) (mg/mL)**	**MIC CLTRI (in the presence of 60 mM AcA) (in mg/mL)**	**MIC FLC (in mg/mL)**	**MIC FLC (in the presence of 40 mM AcA) (in mg/mL)**	**MIC FLC (in the presence of 60 mM AcA) (in mg/mL)**	**MIC CLTRI (in mg/mL)**	**MIC CLTRI (in the presence of 40 mM AcA) (in mg/mL)**	**MIC CLTRI (in the presence of 60 mM AcA) (mg/mL)**
***C. glabrata***												
*C. glabrata* CBS138	32	32	16	8	4	2	8	8	8	1	1	0.5
*C. glabrata* BG2	64	64	16	16	4	2	16	16	32	1	2	2
*C. glabrata* VG99	64	32	16	8	2	1	16	16	16	1	1	1
*C. glabrata* VG281	32	32	16	8	2	1	8	16	16	1	1	1
*C. glabrata* VG216	32	32	16	8	2	1	8	16	16	1	2	2
*C. glabrata* FFUL887 (FLC^R^; K274Q GOF *CgPDR1* allele)	≥64	≥64	64	≥16	16	2	≥64	≥64	≥64	4	4	4
*C. glabrata* FFUL674 (FLC^R^; unknown CgPdr1 GOF *CgPDR1* allele)	≥64	≥64	≥64	≥16	≥16	≥16	≥64	≥64	≥64	4	4	4
*C. glabrata* FFUL677 (FLC^R^; unknown CgPdr1 GOF allele)	≥64	≥64	≥64	≥16	≥16	≥16	≥64	≥64	≥64	8	8	8
*C. glabrata* F15 (FLC^R^; P927L GOF *CgPDR1* allele)	≥64	≥64	≤ 0.25	-	-	-	≥64	≥64	≥64	-	-	-
***C. albicans***												
*C. albicans* SC5314	0.5	≤ 0.25	-	0.1	≤ 0.0125	-	≤ 0.0125	≤ 0.25	-	≤ 0.00625	≤ 0.0125	-
*C. albicans* CaVG216	0.5	≤ 0.25	-	0.1	≤ 0.0125	-	0.025	≤ 0.25	-	≤ 0.00625	≤ 0.0125	-
*C. albicans* CaVG485	0.5	≤ 0.25	-	0.1	≤ 0.0125	-	0.025	≤ 0.25	-	≤ 0.00625	≤ 0.0125	-

*The MIC was determined by the microdilution method, as recommended by EUCAST*.

## Discussion

The interest in the study of the effect on the physiology of *C. albicans* or *C. glabrata* of lactic and acetic acids at a low pH is boosted by the hypothesis that these organic acids play an important role in the control of the overgrowth of vaginal pathogens (Hickey et al., 2012; Aldunate et al., 2015; Petrova et al., 2015; Tachedjian et al., 2017). While various studies have demonstrated a prominent effect of lactic acid at a low pH in inactivating bacterial vaginal pathogens (O'hanlon et al., 2011, 2013), in the case of fungal species this topic has only been marginally investigated. Under the conditions that we have used, physiologically relevant concentrations of acetic acid significantly inhibited growth of *C. albicans* and *C. glabrata*, although at different extents since the latter species was considerably more tolerant. This particularly high susceptibility to acetic acid of *C. albicans* was observed both in lab (SC5314) and in two vaginal strains (Figure 6). Consistent with the idea that it is the undissociated form of acetic acid that induces toxicity against yeast cells, the decrease in pH (which favors the concentration of the undissociated form) led to a stronger inhibitory effect (Figures 1, 6B). Differently, lactic acid exerted no significant effect in inhibiting growth of *C. albicans* or *C. glabrata*, not even at pH 3 that is already well below the acid pKa (~3.9). These results are in agreement with those obtained in prior studies that also reported an absence of lactic acid toxicity against *C. albicans* at acidic pHs (Moosa et al., 2004; Kasper et al., 2015). *C. albicans* was shown to consume lactic acid even when glucose is present in the growth medium and this was also hypothesized to occur for other short-chain fatty acids such as acetate (Childers et al., 2016). In the case of *C. glabrata* lactate and acetate utilization was shown (although lactate was clearly favored) and metabolization of these organic acids in the presence of glucose was also hypothesized (Ueno et al., 2011). More recently, co-consumption of glucose and acetic acid in *C. glabrata* was also shown, although this occurred only when cells were adapted and exponentially growing in the presence of the acid indicating that a prior adaptation response is required (Cunha et al., 2017). The ability of pathogenic *Candida* species to mobilize acetate or lactate even when glucose is present in the environment was suggested to favor virulence by contributing to improve metabolic versatility in infection niches often deprived of glucose such as the vaginal tract (Childers et al., 2016). In this context, it is reasonable to conceive that the low tolerance exhibited by *C. albicans* and *C. glabrata* to lactic acid could result from their rapid ability to fuel it for metabolization, while in the case of acetic acid this may occur at a much slower rate potentiating the toxic effects of the acid. Although *C. albicans* is equipped with an efficient lactate-uptake system also able to mediate acetate uptake (Vieira et al., 2010), and in *C. glabrata* transporters suggested to mediate acetate uptake have also been identified (Mota et al., 2015), at the acidic vaginal pH (close to or below the acids pKa) the entry of acetic and lactic acids is expected to occur by passive diffusion of the undissociated forms. As such, it is unlikely that the activity of these transport systems might play a significant role in modulating tolerance to those organic acids.

The observed lack of inhibitory effect of lactic acid against *C. albicans* and *C. glabrata* species suggests that the reported antimicrobial potential of supernatants obtained from vaginal lactobacilii cultures against *Candida* (e.g., Parolin et al., 2015) could result from other compounds present therein. In line with this hypothesis, metabolomic analysis of vaginal lactobacilii culture supernatants could not correlate the amount of lactic acid with the anti-candicidal potential of the supernatant, although this was encountered for other metabolites (Parolin et al., 2015). Further studies better characterizing the composition of these bacterial supernatants are required to clarify their inhibitory potential against *Candida* spp. The herein observed high toxicity of acetic acid against *C. albicans* and *C. glabrata* also indicates that mechanisms assuring tolerance of these yeast species to cope with this organic acid should play an important role to assure competitiveness and avoid exclusion from the vaginal tract, specially under dysbiosis conditions where the concentration of this organic acid increases prominently (~120 mM) (Aldunate et al., 2015). In *C. glabrata* a few of those adaptive responses have been studied (Bernardo et al., 2017; Cunha et al., 2017) but in *C. albicans* this matter had not been thoroughly examined since the studies undertaken until so far examined responses using almost non-inhibitory conditions at pH 5.5, well above the acid pKa (Cottier et al., 2015, 2017). A particularly interesting aspect of these results would be to assess whether the competitiveness of *C. albicans*, compared to that of *C. glabrata*, in women with bacterial vaginosis, a condition known to increase acetic acid concentration. The metagenomic studies performed until so far of the vaginal micro- and myco- biomes do not allow the establishment of that correlation, either due to lack of identification at the species level or by only performing myco- or micro- biome analysis.

The effect exerted by the presence of acetic and lactic acids in modulating *C. albicans* and *C. glabrata* tolerance to azole drugs was another of the objectives of this work, this being a relevant interaction since these molecules may co-exist in the vaginal tract. It has been reported the reduced effect against *C. albicans* and *C. glabrata* of several antifungals belonging to different classes under acidic pHs using strong acids as the acidulants (Danby et al., 2012) and our study confirms these prior observations (Table 2). The presence of acetic acid improved the efficacy of all azoles tested against *C. albicans*—consistent with the results obtained by Moosa et al. (2004)-while for *C. glabrata* synergism was only observed for clotrimazole and fluconazole and this was strain-dependent. Against *C. glabrata* the synergistic effect was only observed for concentrations of acetic acid equal or above 40 mM (at pH 4), while for *C. albicans* this was observed even at 4 mM of acetic acid (at pH 4), a concentration within the range of those found in vaginal fluid even under eubiosis conditions (Boskey et al., 1999, 2001; Aldunate et al., 2015). This observation is consistent with the higher tolerance of the *C. glabrata* strains to acetic acid alone. Lactic acid improved efficacy of azoles only against *C. albicans* and at concentrations above 80 mM (at pH4), a value in line with those found in vaginal fluid (Boskey et al., 1999, 2001; Aldunate et al., 2015). Interestingly, this synergistic effect between azoles and lactic acid was not uncovered before in the study of Moosa et al. (2004), probably because in this work the authors have only used around 20 mM lactic acid, below concentrations reported to occur in vaginal fluid (Aldunate et al., 2015). One of the reported effects of undissociated organic acids (including of acetic acid) in yeast cells is the perturbation of the plasma membrane structure (Mira et al., 2010) which can thereby facilitate the entry of the azole drug into the yeast cells. Further studies are required to better investigate this synergistic effect between acetic and/or lactic acids and azoles in *Candida* spp. Another important feature of this interaction between organic acids and azoles is the previous demonstration that the combination of these two molecules has a fungicidal effect against *C. albicans* (Moosa et al., 2004), while when used alone the effect is only fungistatic.

One significant observation from our study is that the presence of acetic acid sensitized to azoles *C. glabrata* strains that were described to be resistant to these molecules, albeit not at the same extent since the effect was much more prominent for the F15 strain than for the other strains tested (Figure 6A). This sensitization effect correlated with the higher susceptibility of these strains to acetic acid (Figure 6B). The azole-resistance phenotype of all the *C. glabrata* strains used in our study results from them harboring different gain-of-function (GOF) mutations in the coding sequence of the transcriptional activator CgPdr1(Vermitsky et al., 2006 and our unpublished results; Salazar et al., 2018), an essential determinant of antifungal response in *C. glabrata*. Previously it had been suggested that the high susceptibility of FFUL887 and F15 strains to acetic acid may result from them expressing a CgPdr1 gain-of-function allele (Vermitsky et al., 2006; Salazar et al., 2018), which is in line with the results that we have obtained in the present work. Azole-resistant *C. albicans* strains were also found to be sensitized to fluconazole in the presence of acetic acid, however, in this case the molecular basis of the resistance phenotype of the strains was not investigated and thus no link with CaTac1 activity (the ortholog of CgPdr1) was made (Moosa et al., 2004). On the overall the results obtained in our work show that tolerance to acetic acid should play a relevant contributing role to assure competitiveness of *C. albicans* and *C. glabrata* in the vaginal tract, including under azole therapy. This should be a particularly relevant trait for successful colonization by *C. albicans*, which was found to be highly susceptible to this organic acid. The observed lack of effect of lactic acid in inhibiting growth of *C. albicans* and *C. glabrata* contrasts with the generalized idea that production of this organic acid by vaginal bacteria underlies a protective effect exerted against *Candida* species. It is also rendered clear that the levels of acetic and lactic acid in the vaginal tract may modulate susceptibility of *C. albicans* and *C. glabrata* to azoles used in treatment of vaginal infections, including those caused by some azole-resistant strains; this representing an important knowledge to improve therapeutic success.

## Author Contributions

AL, NP, and SS contributed to the phenotypic screenings conducted in the presence of organic acids under different conditions including in the presence of azoles. NPM coordinated and conceived the study, also having written the manuscript with contributions from NP and SS.

### Conflict of Interest Statement

The authors declare that the research was conducted in the absence of any commercial or financial relationships that could be construed as a potential conflict of interest.
