# Supplementary material for: Effect of Acetic Acid and Lactic Acid at Low pH in Growth and Azole Resistance of Candida albicans and Candida glabrata
Source: Front Microbiol. 2019 Jan 8;9:3265. doi: 10.3389/fmicb.2018.03265 (PMC6331520; doi:10.3389/fmicb.2018.03265)
Supplement: Supplementary file 1 [file Data_Sheet_1.PDF]

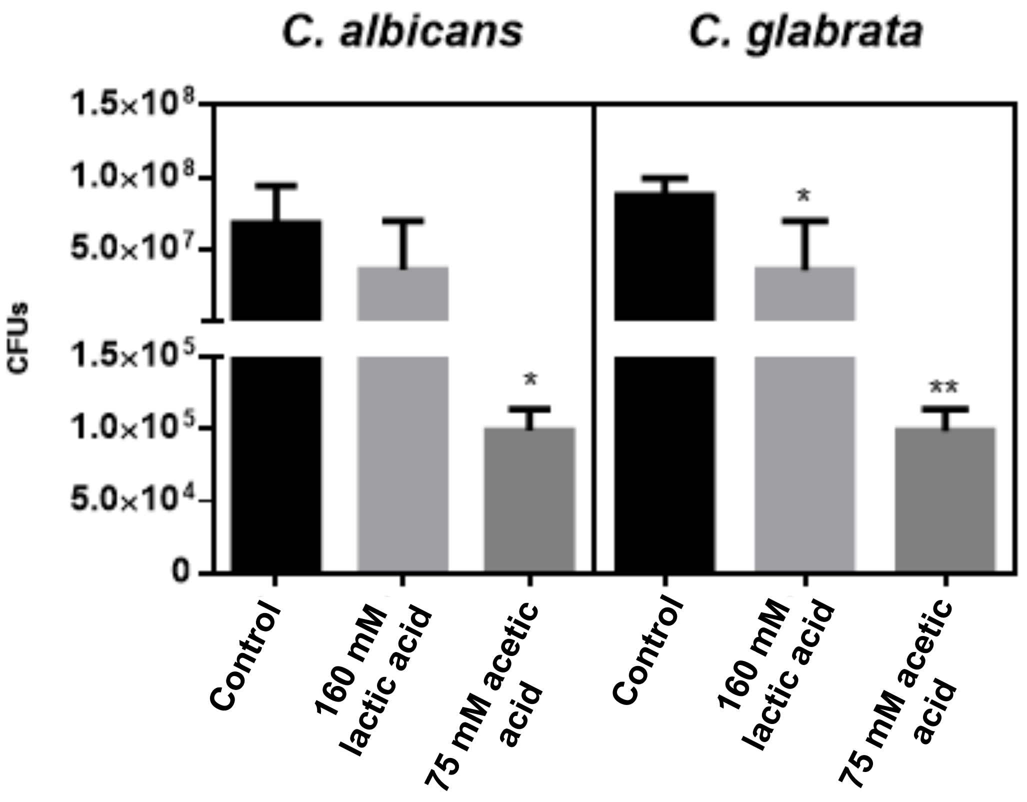

Figure S1. Effect of the presence of lactic acid (160 mM at pH 4) and acetic acid (75 mM at pH 4) in cellular viability of *C. albicans* SC5314 and *C. glabrata* CBS138. Cells of the two yeast species were cultivated for 24h in 96-microwell plates under aerophilic conditions in MM medium supplemented or not with the indicated concentrations of lactic or acetic acid, after which the number of viable cells was estimated based on the formation of CFUs onto the surface of YPD plates (**p*-value below 0.05; ***p*-value below 0.01;)

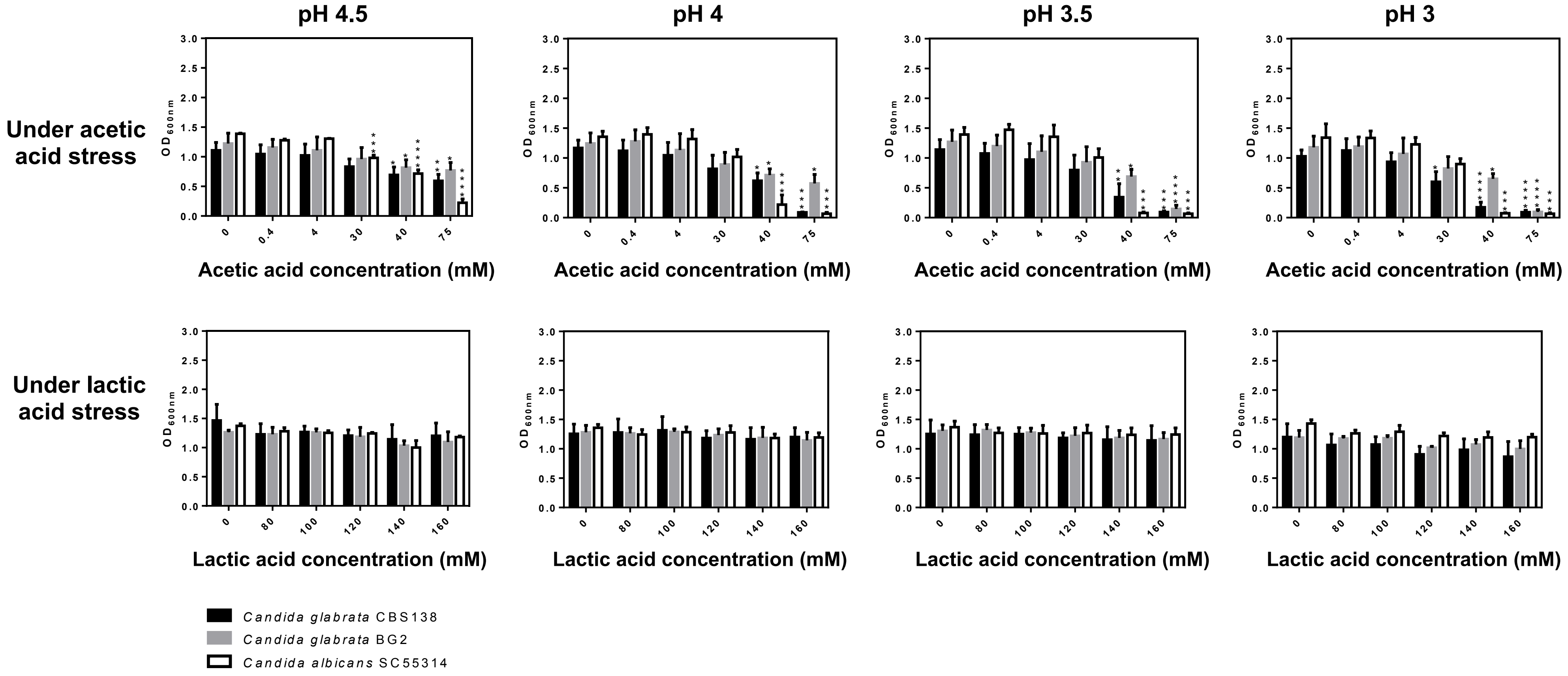

Figure S2. Growth under aerophilic conditions of *C. albicans* (white bars), *C. glabrata* CBS138 (black bars) and *C. glabrata* BG2 (grey bars) in MM medium supplemented with 0.2% glucose and with the indicated concentrations of lactic or acetic acid at the pHs depicted in the figure. Asterisks indicate significance of the difference obtained in comparison with growth observed in the absence of the acid (0 mM, control) (**p*-value below 0.05; ***p*-value below 0.01; ****p*-value below 0.001; **** *p*-value below 0.0001)

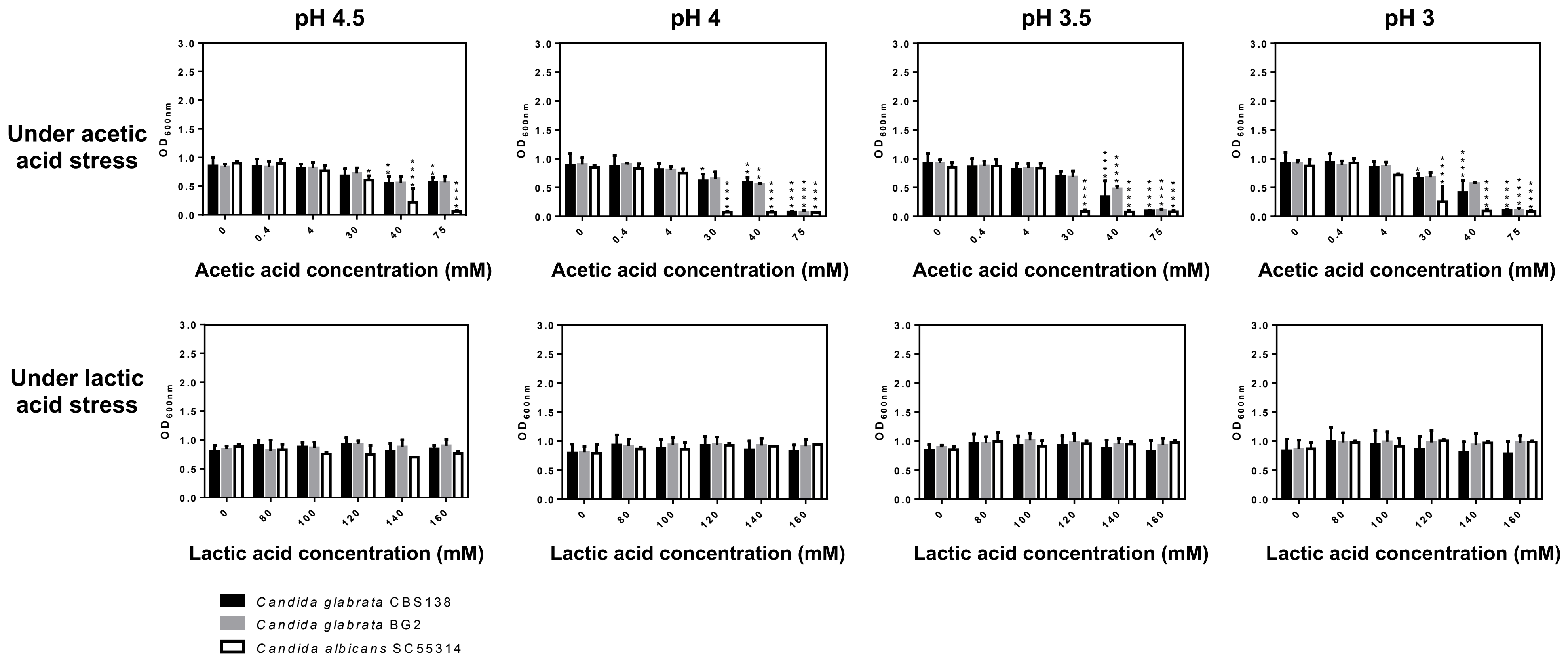

Figure S3. Growth under microaerophilic conditions of *C. albicans* (white bars), *C. glabrata* CBS138 (black bars) and *C. glabrata* BG2 (grey bars) in MM medium supplemented with 0.2% glucose and with the indicated concentrations of lactic or acetic acid at the pHs depicted in the figure. Asterisks indicate significance of the difference obtained in comparison with growth observed in the absence of the acid (0 mM, control) (**p*-value below 0.05; ***p*-value below 0.01; ****p*-value below 0.001; **** *p*-value below 0.0001)

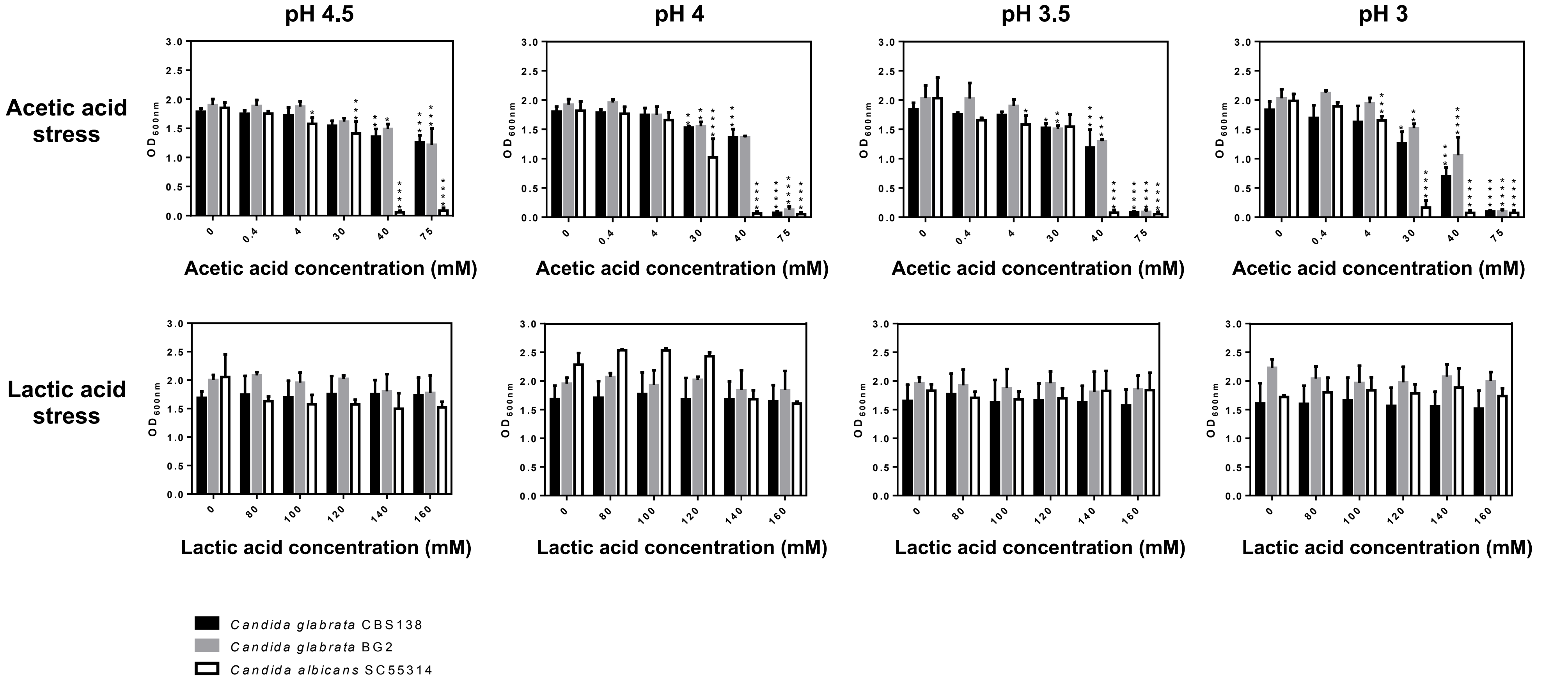

Supplementary figure S4. Growth under aerophilic conditions of *C. albicans* (white bars), *C. glabrata* CBS138 (black bars) and *C. glabrata* BG2 (grey bars) in MM medium (0.2% glucose) supplemented with the indicated concentrations of lactic or acetic acid at the pHs depicted in the figure. Asterisks indicate significance of the difference obtained in comparison with growth observed in the absence of the acid (0 mM, control) (**p*-value below 0.05; ***p*-value below 0.01; ****p*-value below 0.001; **** *p*-value below 0.0001)

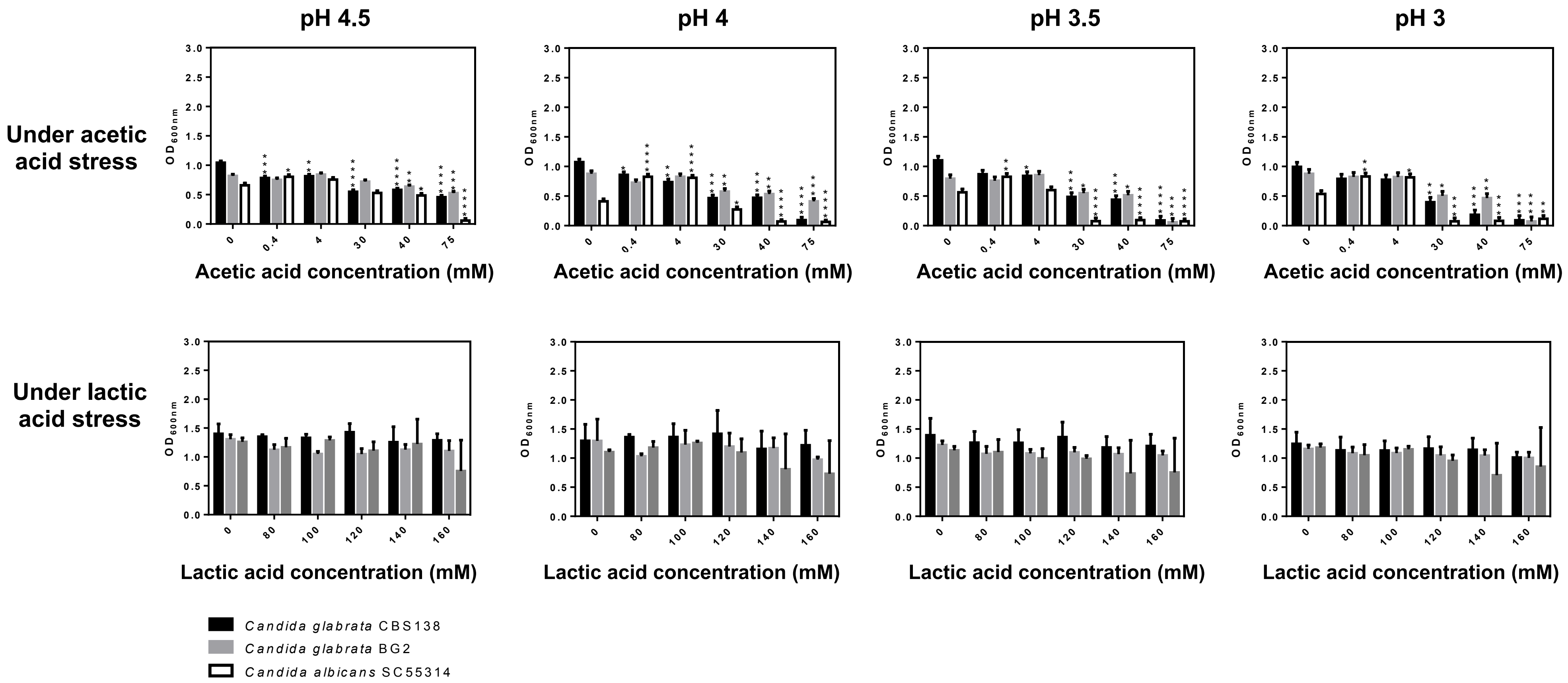

Figure S5. Growth under microaerophilic conditions of *C. albicans* (white bars), *C. glabrata* CBS138 (black bars) and *C. glabrata* BG2 (grey bars) in RPMI medium supplemented with 0.2% glucose and with the indicated concentrations of lactic or acetic acid at the pHs depicted in the figure. Asterisks indicate significance of the difference obtained in comparison with growth observed in the absence of the acid (0 mM, control) (**p*-value below 0.05; ***p*-value below 0.01; ****p*-value below 0.001; **** *p*-value below 0.0001)

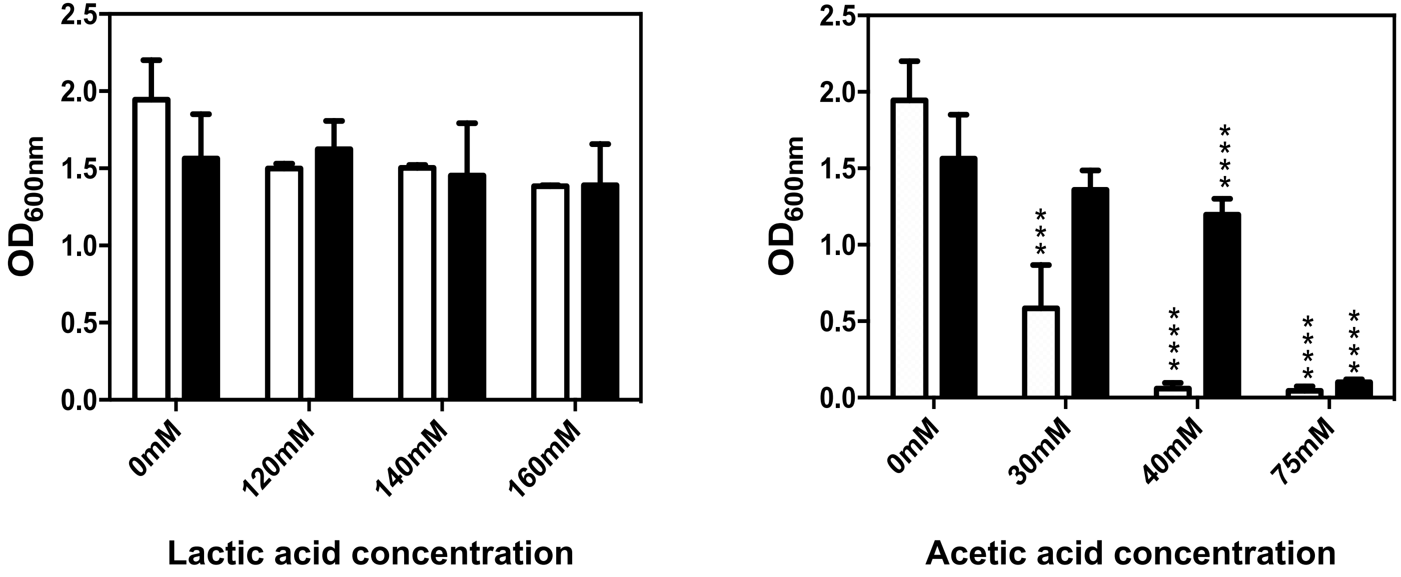

Figure S6. Growth under aerophilic conditions of *C. albicans* (white bars) and *C. glabrata* CBS138 (black bars) and *C. glabrata* BG2 (grey bars) in MM medium supplemented with 1% glucose and with the indicated concentrations of lactic or acetic acid at 37ºC. Asterisks indicate significance of the difference obtained in comparison with growth observed in the absence of the acid (0 mM, control) (**p*-value below 0.05; ***p*-value below 0.01; ****p*-value below 0.001; **** *p*-value below 0.0001)

C. glabrata CBS138

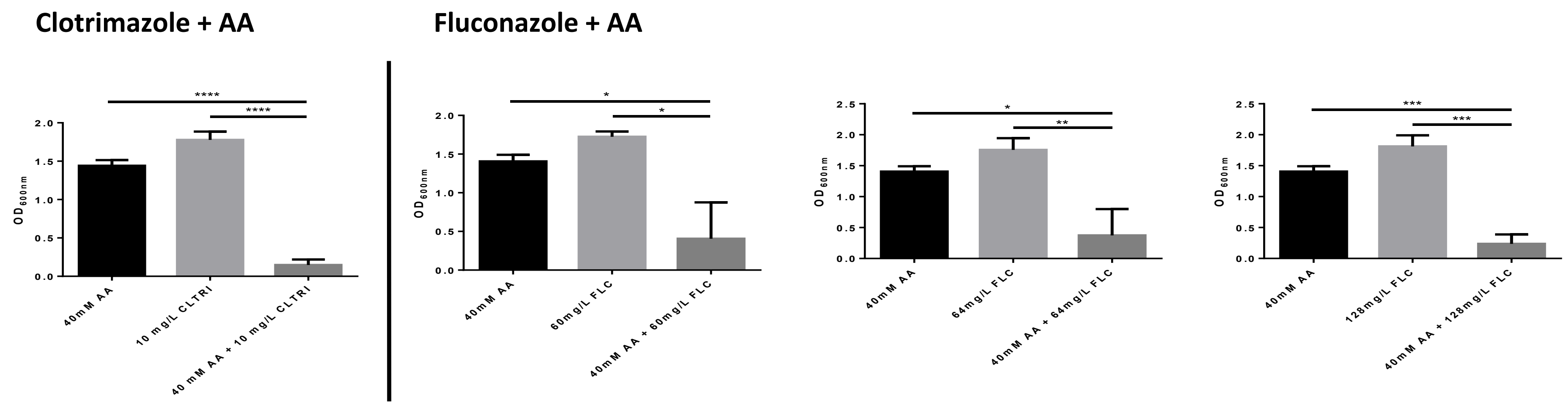

Figure S7. Effect of the presence of acetic acid (AA) and clotrimazole (Clotri) or fluconazole (FLC) alone or in combination in growth of *C. glabrata CBS138* under the experimental conditions defined in the heat-map shown in Fig.4. Herein is presented the ODs obtained after 24h of growth in the presence of the indicated concentrations of the two compounds either alone or in combination. Statistical difference between the growth inhibition obtained using AA and the azoles combined or alone was performed using a two-way Anova test. (**p*-value below 0.05; ***p*-value below 0.01; ****p*-value below 0.001; **** *p*-value below 0.0001)

C. glabrata BG2

Clotrimazole + AA

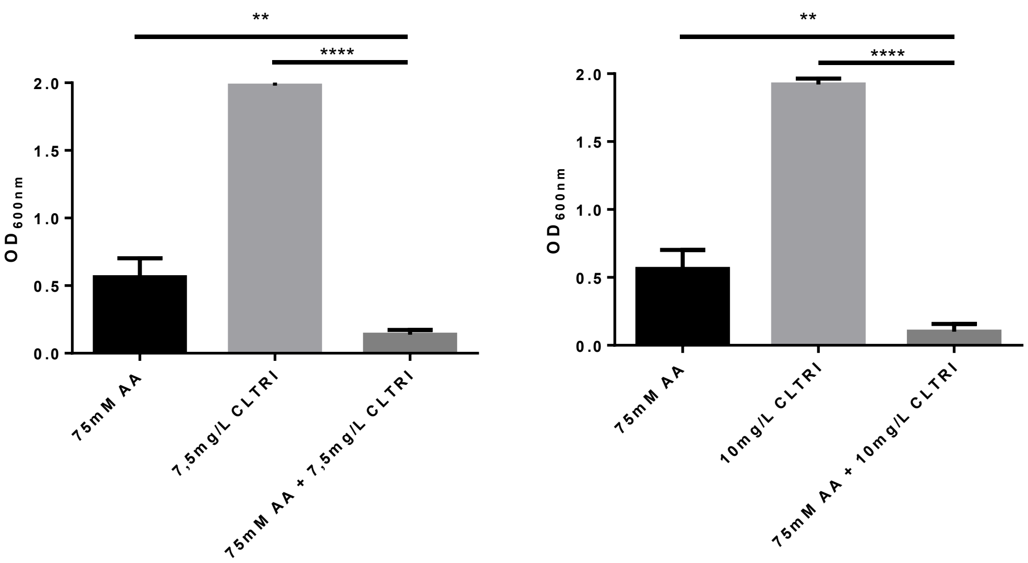

Figure S7. Effect of the presence of acetic acid (AA) and clotrimazole (Clotri) alone or in combination in growth of *C. glabrata* BG2 under the experimental conditions defined in the heat-map shown in Fig.4. Herein is presented the ODs obtained after 24h of growth in the presence of the indicated concentrations of the two compounds either alone or in combination. Statistical difference between the growth inhibition obtained using AA and the azoles combined or alone was performed using a two-way Anova test. (**p*-value below 0.05; ***p*-value below 0.01; ****p*-value below 0.001; **** *p*-value below 0.0001)

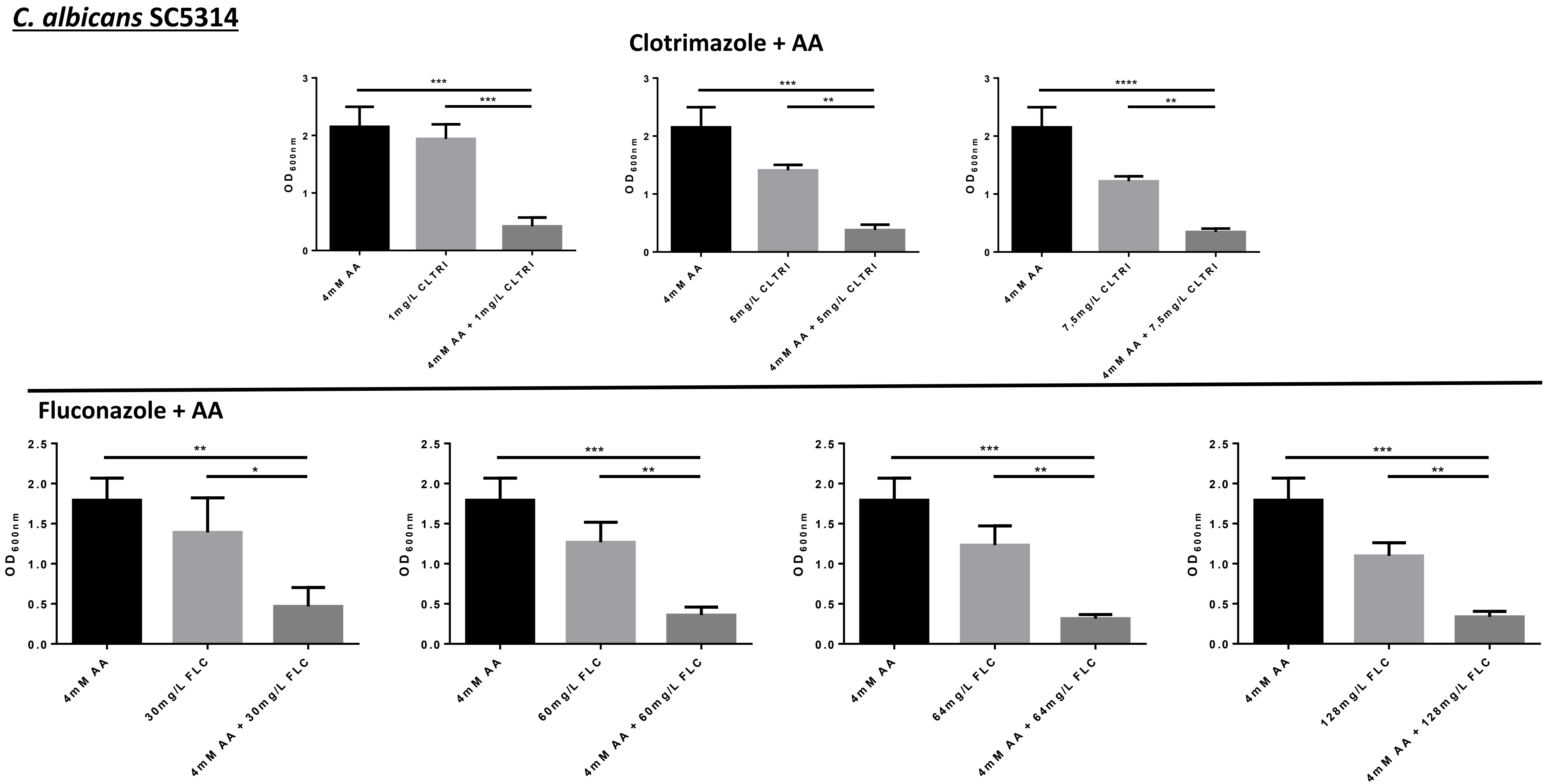

Figure S7. Effect of the presence of acetic acid (AA) and fluconzole (FLC) alone or in combination in growth of *C. albicans* SC5314 under the experimental conditions defined in the heat-map shown in Fig.4. Herein is presented the ODs obtained after 24h of growth in the presence of the indicated concentrations of the two compounds either alone or in combination. Statistical difference between the growth inhibition obtained using AA and the azoles, combined or alone, was performed using a two-way Anova test. (**p*-value below 0.05; ***p*-value below 0.01; ****p*-value below 0.001; **** *p*-value below 0.0001)

C. albicans **SC5314**

Miconazole + AA

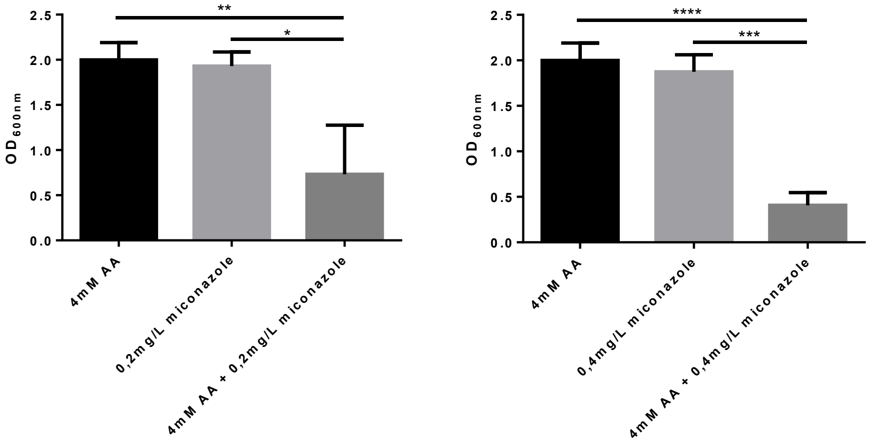

Tioconazole + AA

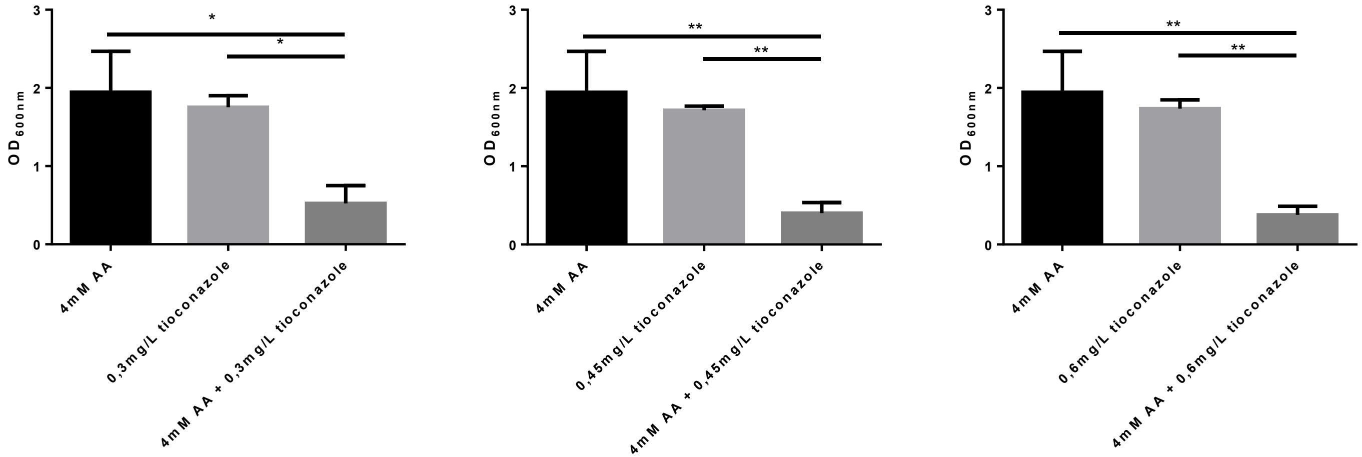

Figure S7. Effect of the presence of acetic acid (AA) and Miconazole or tioconazole alone or in combination in growth of *C. albicans* SC5314 under the experimental conditions defined in the heat-map shown in Fig.4. Herein is presented the ODs obtained after 24h of growth in the presence of the indicated concentrations of the two compounds either alone or in combination. Statistical difference between the growth inhibition obtained using AA and the azoles, combined or alone, was performed using a two-way Anova test. (**p*-value below 0.05; ***p*-value below 0.01; ****p*-value below 0.001; **** *p*-value below 0.0001)

C. albicans SC5314

Clotrimazole + LA

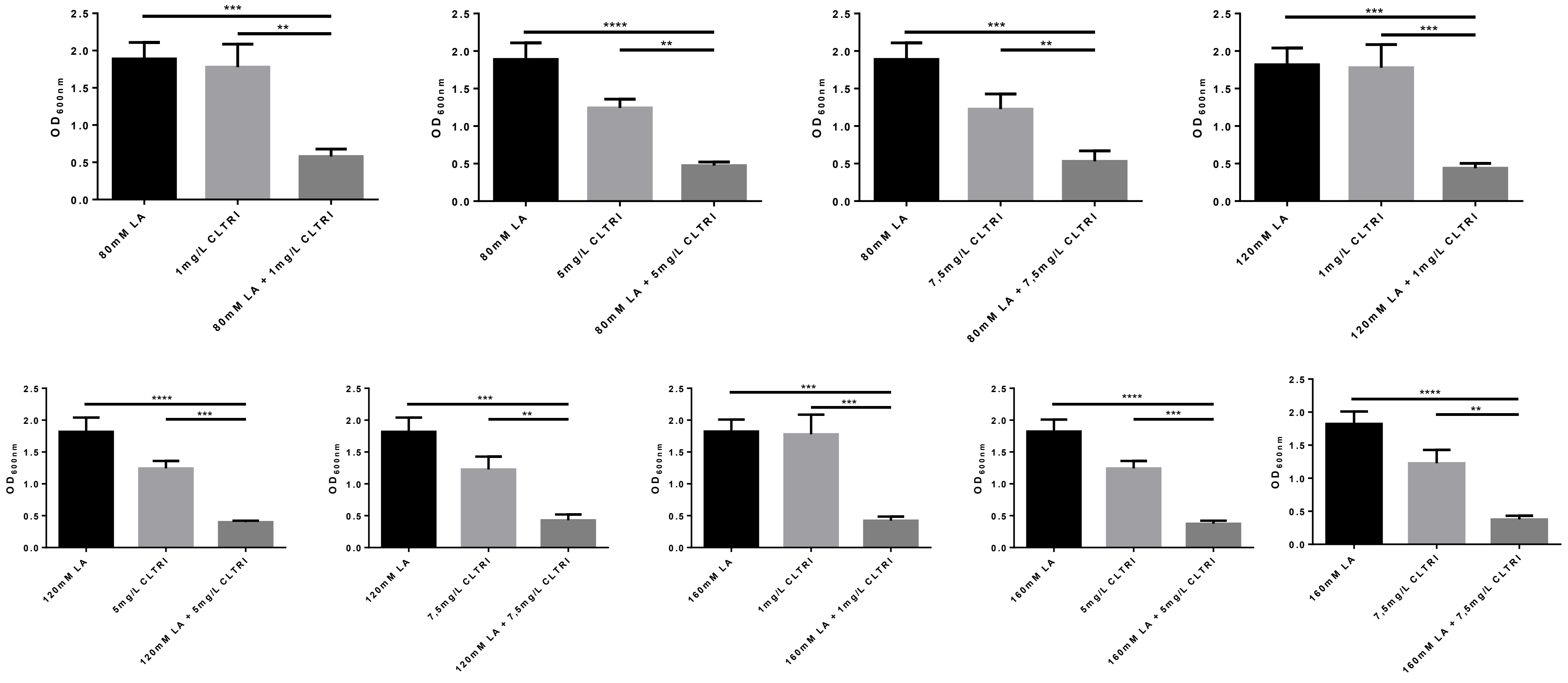

Figure S7. Effect of the presence of lactic acid (LA) and Clotrimazole (clotri) alone or in combination in growth of *C. albicans* SC5314 under the experimental conditions defined in the heat-map shown in Fig.4. Herein is presented the ODs obtained after 24h of growth in the presence of the indicated concentrations of the two compounds either alone or in combination. Statistical difference between the growth inhibition obtained using LA and the azoles, combined or alone, was performed using a two-way Anova test. (**p*-value below 0.05; ***p*-value below 0.01; ****p*-value below 0.001; **** *p*-value below 0.0001)

C. albicans SC5314

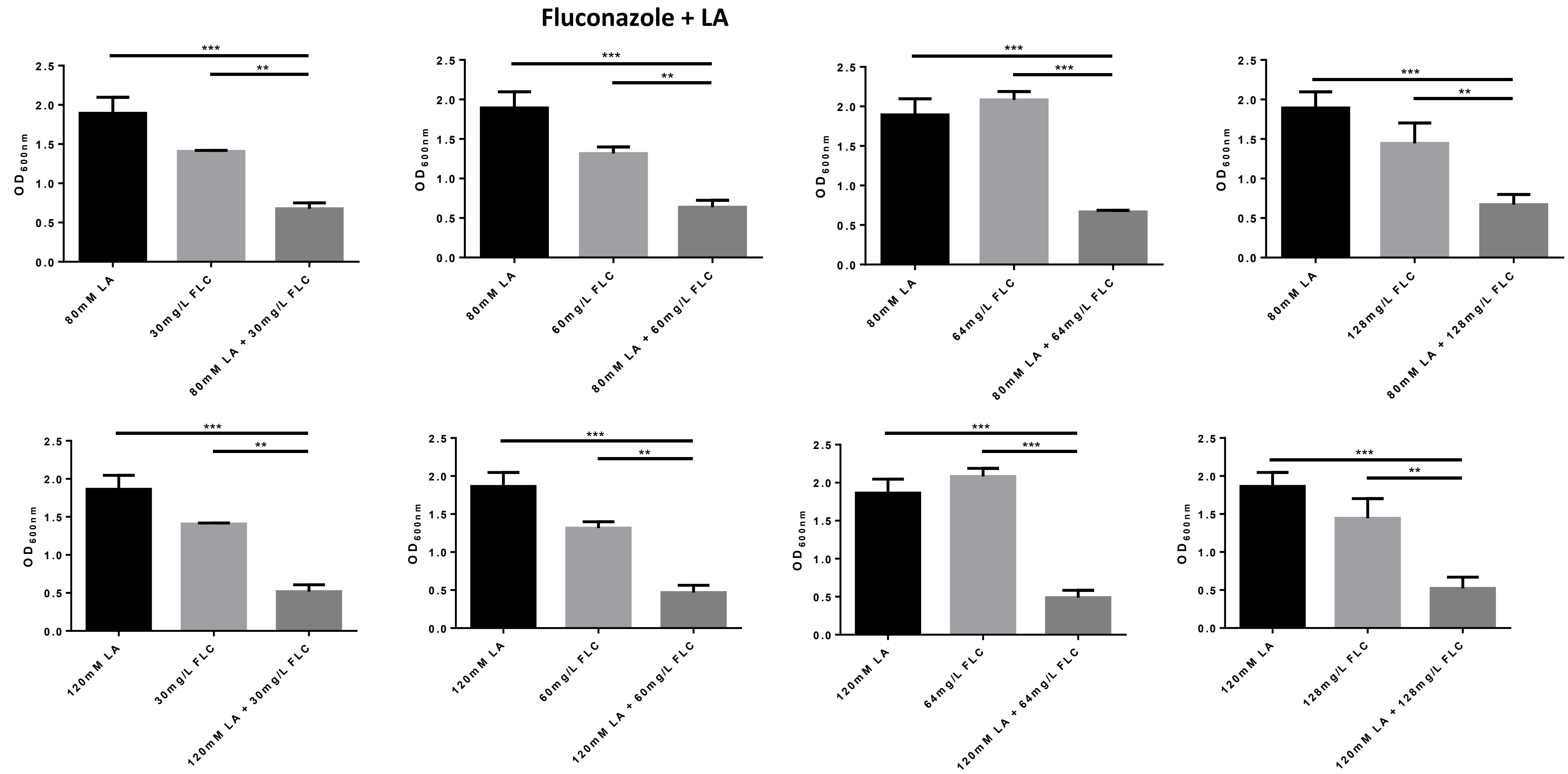

Figure S7. Effect of the presence of lactic acid (LA) and fluconazole (FLC) alone or in combination in growth of *C. albicans* SC5314 under the experimental conditions defined in the heat-map shown in Fig.4. Herein is presented the ODs obtained after 24h of growth in the presence of the indicated concentrations of the two compounds either alone or in combination. Statistical difference between the growth inhibition obtained using LA and the azoles, combined or alone, was performed using a two-way Anova test. (**p*-value below 0.05; ***p*-value below 0.01; ****p*-value below 0.001; **** *p*-value below 0.0001)

C. albicans SC5314

Fluconazole + LA

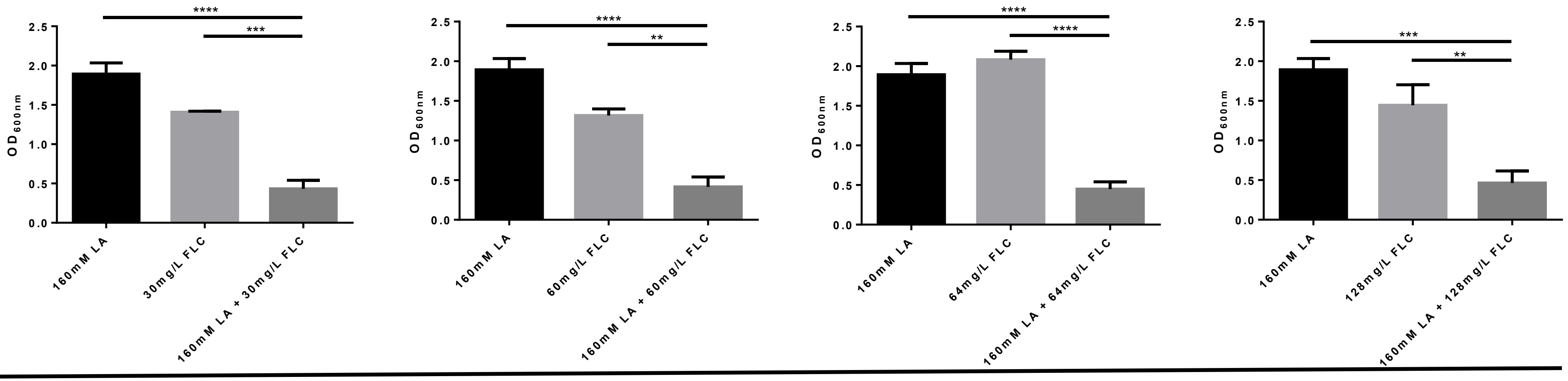

Miconazole + LA

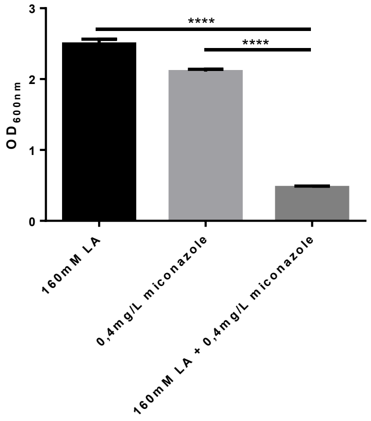

Figure S7. Effect of the presence of lactic acid (LA) and fluconazole (FLC) or Miconazole alone or in combination in growth of *C. albicans* SC5314 under the experimental conditions defined in the heat-map shown in Fig.4. Herein is presented the ODs obtained after 24h of growth in the presence of the indicated concentrations of the two compounds either alone or in combination. Statistical difference between the growth inhibition obtained using LA and the azoles, combined or alone, was performed using a two-way Anova test. (**p*-value below 0.05; ***p*-value below 0.01; ****p*-value below 0.001; **** *p*-value below 0.0001)

***C. albicans* SC5314**

Tioconazole + LA

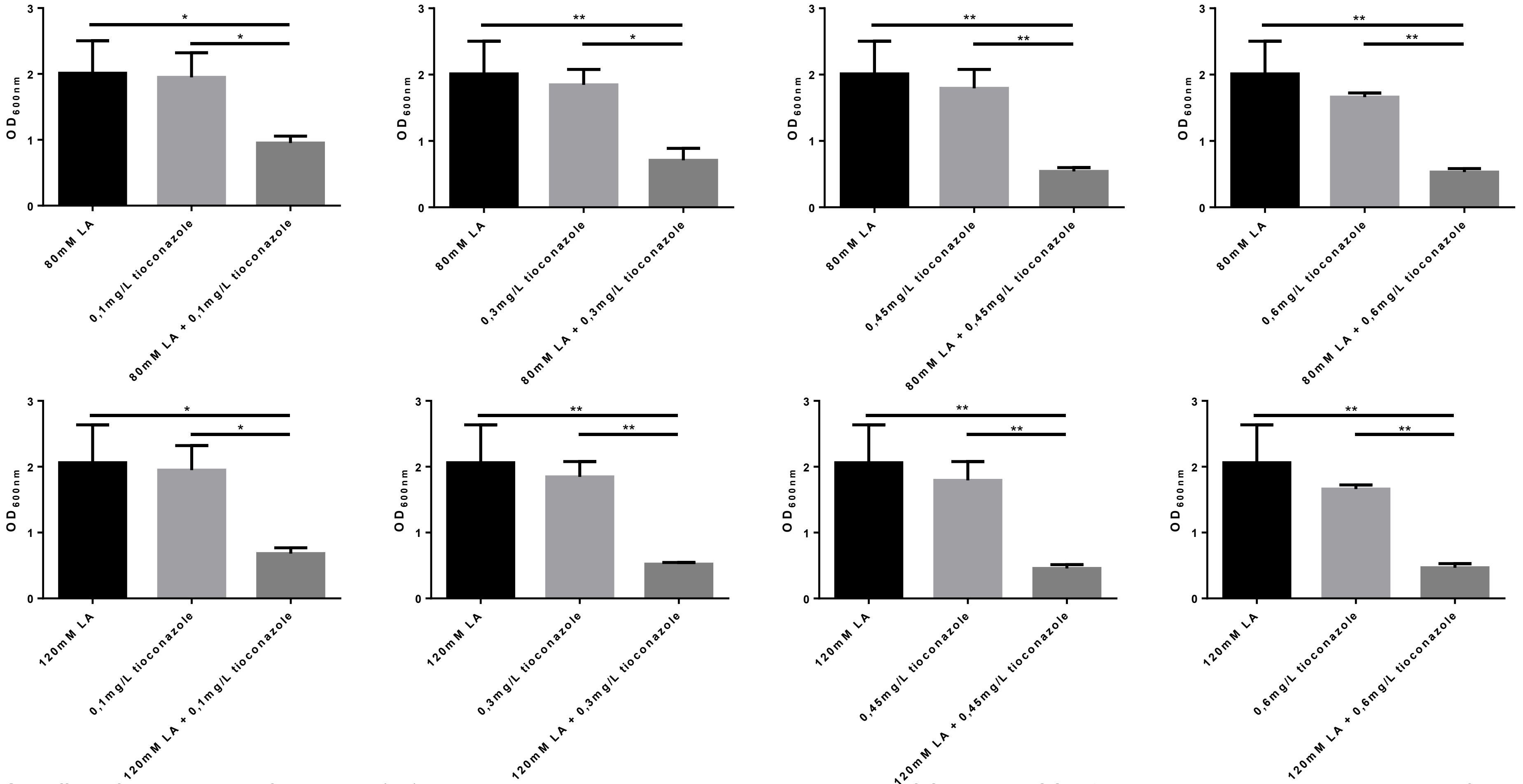

Figure S7. Effect of the presence of lactic acid (LA) and tioconazole alone or in combination in growth of *C. albicans* SC5314 under the experimental conditions defined in the heat-map shown in Fig.4. Herein is presented the ODs obtained after 24h of growth in the presence of the indicated concentrations of the two compounds either alone or in combination. Statistical difference between the growth inhibition obtained using LA and the azoles, combined or alone, was performed using a two-way Anova test. (**p*-value below 0.05; ***p*-value below 0.01; ****p*-value below 0.001; **** *p*-value below 0.0001)

C. albicans SC5314

Tioconazole + LA

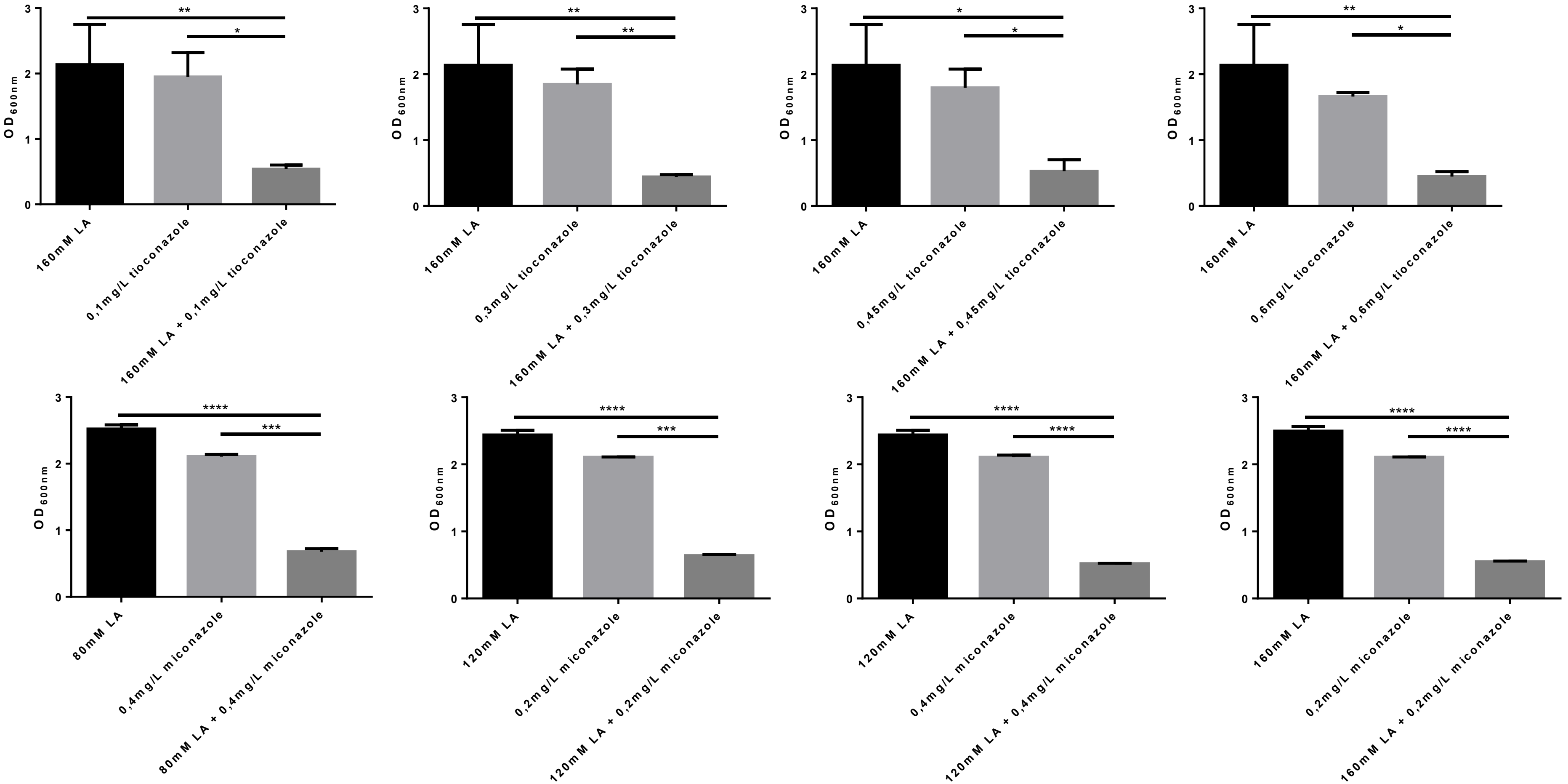

Figure S7. Effect of the presence of lactic acid (LA) and tioconazole alone or in combination in growth of *C. albicans* SC5314 under the experimental conditions defined in the heat-map shown in Fig.4. Herein is presented the ODs obtained after 24h of growth in the presence of the indicated concentrations of the two compounds either alone or in combination. Statistical difference between the growth inhibition obtained using LA and the azoles, combined or alone, was performed using a two-way Anova test. (*p-value below 0.05; **p-value below 0.01; ***p-value below 0.001; **** p-value below 0.0001)
